# Assessment of Interprofessional Collaborative Practices and Outcomes in Adults With Diabetes and Hypertension in Primary Care: A Systematic Review and Meta-analysis

**DOI:** 10.1001/jamanetworkopen.2020.36725

**Published:** 2021-02-12

**Authors:** Jeannie K. Lee, Livia R. M. McCutcheon, Maryam T. Fazel, Janet H. Cooley, Marion K. Slack

**Affiliations:** 1 The University of Arizona College of Pharmacy, Tucson; 2 Star Wellness Family Practice, St Luke’s Family Medicine Residency, Bethlehem, Pennsylvania; 3 Nesbitt School of Pharmacy, Wilkes University, Wilkes-Barre, Pennsylvania

## Abstract

**Importance:**

Interprofessional collaborative practice (ICP), the collaboration of health workers from different professional backgrounds with patients, families, caregivers, and communities, is central to optimal primary care. However, limited evidence exists regarding its association with patient outcomes.

**Objective:**

To examine the association of ICP with hemoglobin A_1C_ (HbA_1c_), systolic blood pressure (SBP), and diastolic blood pressure (DBP) levels among adults receiving primary care.

**Data Sources:**

A literature search of English language journals (January 2013-2018; updated through March 2020) was conducted using MEDLINE; Embase; Ovid IPA; Cochrane Central Register of Controlled Trials: Issue 2 of 12, February 2018; NHS Economic Evaluation Database: Issue 2 of 4, April 2015; Clarivate Analytics WOS Science Citation Index Expanded (1990-2018); EBSCOhost CINAHL Plus With Full Text (1937-2018); Elsevier Scopus; FirstSearch OAIster; AHRQ PCMH Citations Collection; ClinicalTrials.gov; and HSRProj.

**Study Selection:**

Studies needed to evaluate the association of ICP (≥3 professions) with HbA_1c_, SBP, or DBP levels in adults with diabetes and/or hypertension receiving primary care. A dual review was performed for screening and selection.

**Data Extraction and Synthesis:**

This systematic review and meta-analysis followed the PRISMA guideline for data abstractions and Cochrane Collaboration recommendations for bias assessment. Two dual review teams conducted independent data extraction with consensus. Data were pooled using a random-effects model for meta-analyses and forest plots constructed to report standardized mean differences (SMDs). For high heterogeneity (*I*^2^), data were stratified by baseline level and by study design.

**Main Outcomes and Measures:**

The primary outcomes included HbA_1c_, SBP, and DBP levels as determined before data collection.

**Results:**

A total of 3543 titles or abstracts were screened; 170 abstracts or full texts were reviewed. Of 50 articles in the systematic review, 39 (15 randomized clinical trials [RCTs], 24 non-RCTs) were included in the meta-analyses of HbA_1c_ (n = 34), SBP (n = 25), and DBP (n = 24). The sample size ranged from 40 to 20 524, and mean age ranged from 51 to 70 years, with 0% to 100% participants being male. Varied ICP features were reported. The SMD varied by baseline HbA_1c_, although all SMDs significantly favored ICP (HbA_1c_ <8, SMD = −0.13; *P* < .001; HbA_1c_ ≥8  to < 9, SMD = −0.24; *P* = .007; and HbA_1c_ ≥9, SMD = −0.60; *P* < .001). The SMD for SBP and DBP were −0.31 (95% CI, −0.46 to −0.17); *P* < .001 and −0.28 (95% CI, −0.42 to −0.14); *P* < .001, respectively, with effect sizes not associated with baseline levels. Overall *I*^2^ was greater than 80% for all outcomes.

**Conclusions and Relevance:**

This systematic review and meta-analysis found that ICP was associated with reductions in HbA_1c_ regardless of baseline levels as well as with reduced SBP and DBP. However, the greatest reductions were found with HbA_1c_ levels of 9 or higher. The implementation of ICP in primary care may be associated with improvements in patient outcomes in diabetes and hypertension.

## Key Points

**Question:**

Is use of interprofessional collaborative practice (ICP) associated with diabetes and hypertension outcomes in primary care patients?

**Findings:**

In this systematic review and meta-analysis of 39 comparative studies that evaluated interprofessional team care involving 3 or more professions in primary care for adult patients with diabetes and/or hypertension, ICP was associated with improved hemoglobin A_1C_ (HbA_1c_), systolic blood pressure, and diastolic blood pressure levels. Primary care ICP was associated with reductions in HbA_1c_ regardless of baseline levels, but the greatest reductions were found with HbA_1c_ levels of 9 or higher.

**Meaning:**

The findings suggest that implementation of ICP in primary care may be associated with improved clinical outcomes for diabetes and hypertension in adult patients.

## Teaser

This systematic review and meta-analysis assesses interprofessional collaborative practices compared with usual care in primary care patients with diabetes and/or hypertension and hemoglobin A_1C_, systolic blood pressure, and diastolic blood pressure outcomes.

## Introduction

Diabetes and hypertension are substantial causes of heart disease and stroke, which are leading causes of death in the US.^1,2^ In 2018, 34.1 million people (13% of the US population) had diabetes^1^ and 108 million (45% of US adults) had hypertension.^2^ Given the complexity of diabetes and hypertension management, team-based care with physicians, nurses, pharmacists, dietitians, and other health care professionals can be an effective approach.^3,4,5,6^

The World Health Organization defines interprofessional collaborative practice (ICP) as a situation in which “multiple health workers from different professional backgrounds work together with patients, families, carers, and communities to deliver the highest quality of care.”^7^^(p7)^ According to Wagner et al,^8^ the use of ICP is the key to achieving the quadruple aim of “improving patient health, enhancing patient experience, reducing health care costs, and improving the work life of providers and staff.”^8^^(p1)^ Characteristics of ICP teams include shared goals, clarity of roles, effective communication, and shared decision-making.^4,9^

Although ICP is recognized as a central component of providing optimal primary care, to our knowledge, there is limited evidence of its role in patient-oriented health outcomes. Two systematic reviews reported conflicting results for ICP in patients with diabetes.^10,11^ One systematic review of 8 studies showed a nonsignificant reduction in hemoglobin A_1C_ (HbA_1c_) when comparing team-based care with usual care.^10^ In contrast, another review of 7 trials found that team-based care was associated with improved HbA_1c_ levels compared with controls.^11^ A 2019 meta-analysis of 35 studies reported that, compared with usual care, team-based care was associated with improved HbA_1c_, systolic blood pressure (SBP), and diastolic blood pressure (DBP) levels.^6^ This meta-analysis, however, was not a systematic review and included randomized clinical trials (RCTs) only up to 2015 and was not focused on assessing ICP by at least 3 professions in primary care settings.

A previous scoping review (2000-2013) examined the breadth of information on ICP in primary care and reported broad consequences associated with patient outcomes.^12^ This review, without meta-analysis, found 8 studies reporting positive differences in HbA_1c_ and 10 reporting positive differences in BP when ICP was compared with controls. Conversely, 6 additional studies reported no differences in HbA_1c_, and 3 reported no differences in BP.^12^ Therefore, results are mixed in assessing ICP in patients with diabetes and hypertension, and an updated systematic review and meta-analysis is warranted to expand applicable knowledge. Our systematic review and meta-analysis was an extension of the scoping review,^12^ with a literature search updated to 2020 that examined ICP compared with usual care and controls using HbA_1c_, SBP, and DBP in patients with diabetes and/or hypertension receiving primary care.

## Methods

### Study Selection

To be eligible for inclusion in the systematic review, studies had to use a comparative design and evaluate ICP in adults with diabetes and/or hypertension receiving primary care. We selected studies that reported evidence of ICP involving 3 or more health professions; primary care practice; adults having diabetes and/or hypertension; assessment of HbA_1c_, SBP, or DBP levels; and statistical evaluation of ICP. Non-English records, reviews, meta-analyses, drug trials, case studies, editorials, and news articles were excluded. To be included in the meta-analysis, the reported comparative data had to be sufficient to calculate a standardized mean difference (SMD).

#### Definitions for ICP and Primary Care

For the present study, an ICP team was defined as a collaboration among individuals from at least 3 different health professions. At least 1 member of the team needed to serve as the primary care professional bearing the authority to diagnose and initiate treatments.^7,13,14^ Consistent with the previous scoping review, the Starfield definition of primary care was used, which defines primary care as being the first point of entry to a health care system, person focused (not disease oriented), and integrating care from outside professionals.^12,15,16^ The 4 key features of primary care service delivery include access (easy to establish contact with a professional who has gatekeeper roles), longitudinality (timely and complementary patient–health care professional experience), comprehensiveness (meeting a broad range of health needs), and coordination of care (integration of services received from external/specialty health care professionals).^12,15,17^

#### Search Strategy

A systematic search was conducted in March 2018 using resources including MEDLINE; Embase; Ovid IPA; Cochrane Central Register of Controlled Trials: Issue 2 of 12, February 2018; NHS Economic Evaluation Database: Issue 2 of 4, April 2015); Clarivate Analytics WOS Science Citation Index Expanded (1990-2018); EBSCOhost CINAHL Plus With Full Text (1937-2018); Elsevier Scopus; FirstSearch OAIster; AHRQ PCMH Citations Collection; ClinicalTrials.gov; and HSRProj. Results were limited to English and initially to publication years from January 2013 to 2018; this start year was selected to build on the previous scoping review (2000-2013).^12^ A research librarian who participated in the scoping review assisted with our search. The search strategy for MEDLINE is described in eMethods 1 in the Supplement. In addition, an abbreviated search update was performed (2018 to March 2020), using Ovid MEDLINE and Cochrane Library databases.

### Data Collection

This study followed the Preferred Reporting Items for Systematic Reviews and Meta-analyses (PRISMA) reporting guideline for data abstraction in the systematic review.^18,19^ A pharmacist who practices in interprofessional primary care (J.K.L.) led the review and data collection. A dual review process, having 2 teams of 2 reviewers, was used for study inclusion and data extraction using previously tested standardized forms to minimize variability. Each reviewer independently screened articles and extracted data, then met to reconcile the differences by consensus. We collected study characteristics; participant characteristics; team makeup, features, and functions; and clinical outcomes of HbA_1c_, SBP, and DBP.

### Outcomes and Data Analysis

The data for primary outcomes (HbA_1c_, SBP, and DBP) were analyzed separately. The SMD (outcome measure that indicated the difference in effect between ICP and comparison) was calculated for each study. Subsequently, the SMDs were pooled using a random-effects model, and a forest plot was constructed. The SMD provided an overall effect estimate of the ICP. The size of the SMD is considered as small (<0.2), moderate (0.2–0.8), or large (>0.8).^20^ For each outcome, a fail-safe N was calculated to determine the number of studies with no difference required to change a significant result to no difference. The *I*^2^, which measures the percent of variation owing to factors other than random variation, was used to determine whether excessive nonrandom variation was present. Presence of publication bias was evaluated using a funnel plot and Kendall τ rank correlation.

The studies were stratified by design (RCT, prospective cohort, retrospective cohort, and pre-post studies), and the analysis was repeated to determine whether the SMD was associated with study design. For HbA_1c_, stratification by baseline HbA_1c_ was performed to identify associations of ICP with patient cohorts having varied diabetes control status.^21^ In addition, the leave-one-out method was conducted to determine whether specific studies had a substantial role in the pooled SMD. Data analysis was conducted using Comprehensive Meta-Analysis (CMA) software (Biostat Inc). The CIs reported in CMA were corrected using the method of Hartung-Knapp-Sidik-Jonkman.^22^ The a priori *P* value was .05. The meta-analysis process and data are shown in eMethods 2 in the Supplement.

#### Risk of Bias Assessment

Because we included diverse study designs, the tools based on the framework of the Cochrane Collaboration recommendations for Effective Practice and Organization of Care were used.^23^ These tools were developed for bias assessment of RCTs, non-RCT cohorts, and pre-post studies. Each item was ranked low risk of bias, unclear, or high risk of bias. A dual review was performed with consensus generation.

## Results

### Study Selection and Characteristics

**Figure 1.**
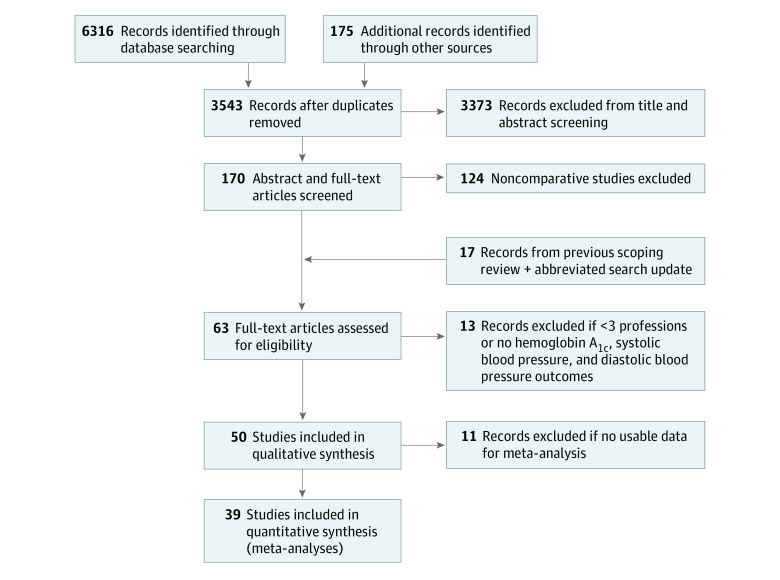
Preferred Reporting Items for Systematic Reviews and Meta-analyses (PRISMA) Flow Diagram for Meta-analyses Inclusion

We identified 6316 articles from the 2013-2018 searches and 175 articles from other sources. After removing duplicates, the review teams screened 3543 titles or abstracts then reviewed 170 abstracts or full-texts to assess 63 articles for eligibility, including the 12 relevant articles from the previous scoping review^12^ and 5 from the abbreviated search update (2018 to March 2020). Of these, 13 records were excluded for having 3 or fewer health professions or no usable outcome measures, leaving 50 articles retained in the systematic review. A final 39 studies were included in the meta-analysis^24,25,26,27,28,29,30,31,32,33,34,35,36,37,38,39,40,41,42,43,44,45,46,47,48,49,50,51,52,53,54,55,56,57,58,59,60,61,62^ after 11 studies were excluded because of inadequate data (eTable in the Supplement).^63,64,65,66,67,68,69,70,71,72,73^ Figure 1 depicts the inclusion process of the systematic review and meta-analysis.

Characteristics of the 50 studies included in the systematic review are listed in Table 1. Of the 39 studies included in the meta-analyses, 15 were RCTs,^24,25,26,27,28,29,30,31,32,33,34,35,36,37,38^ 7 were prospective cohort trials,^39,40,41,42,43,44,45^ 1 was a retrospective cohort,^46^ and 16 were pre-post studies.^47,48,49,50,51,52,53,54,55,56,57,58,59,60,61,62^ Sample size ranged from 40 to 20 524, and study duration ranged from 3 to 24 months. Among the studies that reported patient age and sex, the mean age ranged from 51 to 70 years, and the percentage of male participants ranged from 0 to 100. Studies were most often conducted in the US (n = 18), followed by Brazil (n = 4) and Canada (n = 4), and in an ambulatory care clinic/center/office (n = 9) and community health centers (n = 8). Table 1 also lists ICP team members, roles, main features/process, name of intervention program/model if specified, and other notable intervention details. The team makeup varied widely from the number of professionals involved to types of professions included (3-10). Most teams involved physicians as primary care professionals (n = 36), and most often included professionals from nutrition (n = 33), nursing (n = 32), and pharmacy (n = 20). Similarly, interprofessional team function and intervention features reported by the included studies varied.

**Table 1.** Characteristics of Included Studies in the Meta-analysis

Source	Study design	Setting	Total No.^a^	Age, mean (SD), y	Male, %	Duration, mo	Outcome measures	Team members (No. of professions in team)^b^	Main ICP team features or process (name of intervention program/model if specified OR other notable specifics)^c^
**Randomized clinical trials**									
Barceló et al,^24^ 2010 Mexico	RCT^d^	Public health centers	307	Not reported	Not reported	18	HbA_1c_^e^	Physician; case management advisor nurse; nutritionist and psychologist (at some sites) (4)	Group educational sessions; colocation; visitation by case management advisor (before and after ACIC questionnaire adapted for diabetes)^f^^,^^g^
							SBP^e^		
							DBP^e^		
Cezaretto et al,^25^ 2012 Brazil	RCT^h^	Public health system	177	I: 56.1 (11.4)	32.2	9	SBP^e^	Physician; endocrinologist; nutritionist; psychologist; physical educator (4)	Shared medical appointments and group visits; Biweekly meetings; colocation; face-to-face communication (psycho-educative sessions)^f^^,^^g^
				C: 53.8 (13.3)			DBP^e^		
							FBG		
Cohen et al,^26^ 2011 United States	RCT^h^	Veterans Affairs Health System	99	I: 69.8 (10.7)	I: 100	6	HbA_1c_^e^	Physician; dietitian; nurse; pharmacist; physical therapist (5)	CPA; joint group educational sessions; shared EMR; colocation; face-to-face communication (MEDIC-E)^f^^,^^g^^,^^i^^,^^j^^,^^k^
				C: 67.2 (9.4)	C: 96		SBP^e^		
DePue et al,^27^ 2013 American Samoa	RCT^l^	Community health center	268	55 (12.7)	38	12	HbA_1c_^e^	Physician; CHW; nurse (3)	Medical chart; colocation^f^^,^^k^
							SBP^e^		
							DBP^e^		
Edelman et al,^28^ 2010 United States	RCT^h^	Veterans Affairs Health System	239	I: 63.0 (9.4)	I: 95.5	12.8	HbA_1c_^e^	Physician; nurse or certified diabetes educator; pharmacist (3)	Shared medical appointments and group visits; joint group educational sessions; colocation
				C: 60.8 (10.0)	C: 96.2		SBP^e^		
							DBP^e^		
Goyer et al,^29^ 2013 Canada	RCT^h^	Specialized clinic	185	54.4 (8.6)	I: 64.5	24	HbA_1c_^e^	Physician; kinesiologist; nurse; nutritionist; CV prevention specialist; psychologist (6)	Shared medical appointments or group visits; biweekly meetings; colocation; face-to-face communication^f^^,^^g^
							SBP^e^		
					C: 71		DBP^e^		
							ASCVD risk		
Liou et al,^30^ 2014 Taiwan	RCT^h^	Community health center in underserved areas	95	I: 56.6 (7.7)	I: 52	6	HbA_1c_^e^	Physician; diabetes specialist; dietitian; nurse (4)	Shared medical appointments or group visits via interactive videoconference^f^^,^^g^
				C: 57.0 (7.5)	C: 49		SBP^e^		
							DBP^e^		
Maislos and Weisman,^31^ 2004 Israel	RCT^h^	Mobile clinic	82	I: 58 (14)	I: 24	6	HbA_1c_^e^	Physician; diabetes specialist; dietitian; diabetes educator nurse (3)	Shared medical appointments; colocation; face-to-face communication; shared EMR; treatment protocol^f^^,^^g^^,^^i^^,^^j^^,^^k^
				C: 63 (9)	C: 12				
Pimazoni-Netto et al,^32^ 2011 Brazil	RCT^h^	Ambulatory care clinic/center/office	63	I: 54.5 (1.7)	I: 28	3	HbA_1c_^e^	Physician; diabetes educator; diabetes nurse; exercise trainer; psychologist (5)	Shared medical appointments; colocation; face-to-face communication^f^^,^^i^^,^^j^
				C: 58.4 (1.7)	C: 29				
Ramli et al,^33^ 2016 Malaysia	RCT^d^	Public ambulatory care clinics	888	I: 58 (0.5)	I: 38.2	12	HbA_1c_^e^	Physician; dietitian; medical assistant; medical officer; nurse; nutritionist; pharmacist (7)	Joint or group educational sessions; colocation (EMPOWER-PAR)^f^^,^^g^^,^^i^^,^^j^^,^^k^
				C: 57 (0.5)	C: 35.7		SBP^e^		
							DBP^e^		
Siqueira-Catania et al,^34^ 2013 Brazil	RCT^h^	Primary care center	180	54.7 (12.3)	35	9	SBP^e^	Physician; nutritionist; psychologist; physical educator (4)	Joint or group educational sessions with patients; colocation^f^^,^^g^^,^^j^
							DBP^e^		
Tang et al,^35^ 2013 United States	RCT^h^	Nonprofit health care organization ambulatory care sites	415	I: 54 (10.7)	58.9	12	HbA_1c_^e^	Physician; dietitian; nurse case manager; pharmacist specialist; research assistant (5)	CPA; shared EMR; online messaging (use of wireless glucometers and uploading from home; personalized text and educational videos)^f^^,^^g^^,^^i^^,^^j^
							SBP^e^		
				C: 53.5 (10.2)			DBP^e^		
							ASCVD risk		
Taveira et al,^36^ 2010 United States	RCT^h^	Veteran Affairs Health System	109	I: 62.2 (10.3)	I: 91.4	4	HbA_1c_^e^	Pharmacist (CDE); nurse; nutritionist; physical therapist (4)	Shared medical appointments and group visits; colocation (VA-MEDIC)^f^^,^^g^^,^^i^^,^^j^
				C: 66.8 (10.2)	C: 100		SBP^e^		
							DBP^e^		
Taylor et al,^37^ 2005 Canada	RCT^h^	Ambulatory care clinic	40	I: 58	I: 65	4	HbA_1c_^e^	Physician; dietitian; exercise specialist; nurse care manager and educator (4)	Colocation; nurse worked in a collaborative manner as a case manager, educator, and support person (connecting, empowering, doing for, and finding meaning)^f^^,^^g^
							SBP^e^		
				C: 67	C: 68		DBP^e^		
							FBG		
Tourkmani, et al^38^ 2018 Saudi Arabia	RCT^h^	Chronic diseases center specialized clinics	289	I: 56.9 (12)	I: 34.4	9	HbA_1c_^e^	Senior family physician; clinical pharmacy specialist; dietitian; diabetes educator; health educator; social worker (6)	Weekly team meetings to review patient eligibility and care plans for those already enrolled; colocation; face-to-face communication^f^^,^^i^^,^^j^^,^^k^
				C: 57.5 (11.6)	C: 36.8		SBP^e^		
							DBP^e^		
**Prospective cohort studies**									
Bray et al,^39^ 2013 United States	Prospective cohort	Rural fee-for-service model	727	I: 59.5 (12)	34	18^m^ & 36	HbA_1c_^e^	Physician; dietitian; nurse care manager; pharmacist (4)	Colocation^f^^,^^i^^,^^j^
				C: 60.6 (12.4)			SBP^e^		
							DBP^e^		
Cueto-Manzano et al,^40^ 2013 Mexico	Prospective cohort	Family Medicine Unit - Mexican Institute of Social Security	96	I: 62 (11)	I: 53	6	HbA_1c_^e^	Family physician; dietitian; physical trainer; social worker (4)	Group educational sessions for 2 h per week over 4 wk; colocation^f^^,^^g^
				C: 61 (10)	C: 57		SBP^e^		
							DBP^e^		
Jiao et al,^41^ 2014 Hong Kong	Prospective cohort	Public general outpatient clinics	2144	I: 64.3 (10.9)	I: 49.8	12	HbA_1c_^e^	Physician; advanced practice nurse; consultant in family medicine; dietitian; nurse; optometrist; podiatrist; physiotherapist (8)	Colocation (risk-stratified interventions)^f^^,^^j^
							SBP^e^		
				C: 65.3 (11.7)	C: 49.8		DBP^e^		
							CV event		
Majumdar et al,^42^ 2003 Canada	Prospective cohort	Ambulatory care clinic/center/office	393	I: 63.9 (12.7)	I: 48.7	6	HbA_1c_^e^	Physician; dietitian; nurse diabetes educator; pharmacist; specialist (unspecified) (4)	Group IP educational sessions; monthly educational sessions by traveling team of specialist intervention team; colocation (specialist-to-rural primary care physicians academic detailing)^f^^,^^j^
				C: 21 (12.4)	C: 37.6		SBP		
							DBP		
Panattoni et al,^43^ 2017 United States	Prospective cohort	Nonprofit multidisciplinary group clinics	11 190	I: 56.82	I: 55.54	12	HbA_1c_	Physician; health coach; nurse care manager; unlicensed medical assistant; pharmacist (5)	Joint or group IP educational sessions; shared EMR; colocation; face-to-face communication (champion standard work)^f^^,^^g^^,^^i^^,^^j^^,^^k^
				C: 61.89	C: 50.19		SBP^e^		
							DBP^e^		
Parker et al,^44^ 2016 United States	Prospective cohort	FQCHC	120	I: 52 (8)	I: 32	Project: 36	HbA_1c_^e^	Nurse practitioner/physician; registered nurse; students and faculty from optometry; pharmacist; nurse; health care administrator; physical therapist (7)	Face-to-face communication; joint or group educational sessions; team conferences, including an initial team assessment meeting
				C: 52 (12)	C: 34	Results: from 1 calendar year			
Schouten et al,^45^ 2010 the Netherlands	Prospective cohort	Ambulatory care clinics	1861	I: 66 (12.1)	I: 54.8	12	HbA_1c_^e^	Physician; diabetes nurse; diabetes educator; dietitian; endocrinologist (4)	Joint or group IP educational sessions; consultant services from endocrinologist and diabetes educator; face-to-face communication (quality improvement collaborative)^f^^,^^i^
				C: 67 (11.2)	C: 52.2		SBP^e^		
							DBP^e^		
**Retrospective cohort study**									
Yu et al,^46^ 2017 Hong Kong	Retrospective cohort	Public primary care clinics	20 524	I: 63.8 (9.6)	I: 43.6	12	SBP^e^	Physician; dietitian; nurse; physiotherapist and/or occupational therapist (4 or 5)	Shared EMR; telecommunication/telemedicine; risk assessment and management plan with defined roles for each health care professional–referral process by the care manager^g^^,^^j^^,^^k^
				C: 63.7 (10.0)	C: 43.7		DBP^e^		
**Prospective pre-post studies**									
Collier and Baker,^47^ 2014 United States	Prospective pre-post	Veteran Affairs Health System	138	64.1 (8.6)	Not reported	3	HbA_1c_^e^	Pharmacist (CDE); endocrinologist; health technician clerk; nurse educator; dietitian (5)	PCMH; colocation; shared EMR and CPA (PACT)^j^^,^^k^
Didier and Guimarães,^48^ 2007 Brazil	Prospective pre-post	Health center outpatient service	88	58.03 (9.90)	22	12	SBP^e^	Cardiologist; nurse; nursing technician; nutritionist; social assistant (4)	Joint or group IP educational sessions; shared medical appointments; colocation^f^^,^^g^^,^^i^^,^^j^^,^^k^
							DBP		
**Retrospective pre-post studies**									
Al Asmary et al,^49^ 2013 Saudi Arabia	Retrospective pre-post	Ambulatory care center	41	56.2 (12.9)	41.5	6	HbA_1c_^e^	Physician; diabetes educator; dietitian; health educator; nurse; pharmacist specialist; social worker (7)	Weekly team meetings/rounds/huddles; colocation^f^^,^^g^^,^^i^^,^^j^^,^^k^
							SBP^e^		
							DBP^e^		
							FBG		
BeLue et al,^50^ 2014 United States	Retrospective pre-post	Community health center	189	51	50	24	HbA_1c_^e^	Family medicine physician; dentist; nutritionist; optometrist; psychologist (5)	Colocation, with some members contracted from local hospital/health center (EMPOWER-PAR)^f^^,^^g^^,^^j^
Chwastiak et al,^51^ 2017 United States	Retrospective pre-post	Safety net clinic as part of an academic hospital	634	I: 53.6 (10.3)	I: 60.9	18	HbA_1c_^e^	Primary care professional; care manager (filled by 2 registered nurses, an advanced practice nurse, and a registered dietitian CDE); medical consultant; medical assistant; psychiatric consultant (6)	Weekly caseload review; caseload tracked and maintained by medical assistant (care coordinators were coordinating with outside agencies, and specialist in substance abuse as needed)^f^^,^^j^^,^^k^
				C: 54.7 (11.4)	C: 60.7		SBP		
Deichmann et al,^52^ 2013 United States	Retrospective pre-post	Ochsner Medical Center Outpatient Internal Medicine Clinic	216	Not reported	Not reported	6	HbA_1c_^e^	Physician or nurse practitioner; dietitian; nurse/health coach; pharmacist (4)	Shared EMR; colocation
									Health care professionals saw patients separately during a 2-h visit, 30 min for each rotation through the room
									Nurse coordinator managed the time and transition between health care professionals
Farrell et al,^53^ 2013 United States	Retrospective pre-post	Ambulatory care clinics in an ACO health care network	1032	59.5 (11.9)	47.4	12	HbA_1c_^e^	Physician; BH specialist; care coordinator; pharmacist; registered nurse (mostly CDE) (5)	CPA; shared EMRs; weekly team meetings/rounds/huddles; colocation; face-to-face communication (DDMP)^f^^,^^g^^,^^i^^,^^j^
Gilstrap et al,^54^ 2013 United States	Retrospective pre-post	Community health center	64	51.3	0	24	HbA_1c_^e^	Physician; cardiologist; dietitian; health coach; physical therapist (4)	Colocation (HAPPY Heart Program)^f^^,^^g^
							SBP^e^		
							DBP^e^		
Hassaballa et al,^55^ 2015 United States	Retrospective pre-post	FQCHC	148	Not reported	0	16	HbA_1c_^e^	Physician; BH specialist; CDE; diabetes nurse case manager; diabetes health ambassador (CHW); dietitian; nurse high-risk case manager; outreach nurse; patient navigator; pharmacist specialist; program manager; quality assurance manager (10)	Joint or group educational sessions; PCMH model; shared medical appointments or group visits; colocation (DCCP)^f^^,^^g^^,^^j^
							SBP		
							DBP^e^		
Martin et al,^56^ 2015 United States	Retrospective pre-post	Community health center	48	Not reported	Not reported	About 8.3, average of 250 d	HbA_1c_^e^	Nurse practitioner/physician; nurse; pharmacists; pharmacy students under the supervision of pharmacist preceptors; registered dietitian (4)	Colocation; face-to-face communication shared medical appointments (in a single visit, the patient met with the nurse, then the pharmacy students, then the dietitian, and in addition the physician or nurse practitioner; health care professionals met at the end of clinic day and discussed patients and set follow-up.)
Moinfar et al,^57^ 2016 Iran	Retrospective pre-post	Ambulatory care centers (academic institution)	435	56.5 (9.7)	3	3	HbA_1c_^e^	Physician; nurse; nutritionist; psychologist could be consulted (4)	Colocation; IP group educational sessions on diagnosis and management of psychiatric disorders; use of practice guidelines developed in consultation with specialist physicians in consensus panel sessions^f^^,^^i^^,^^j^
							SBP^e^		
							DBP^e^		
							FBG		
Nagelkerk et al,^58^ 2018 United States	Retrospective pre-post	FQCHC	250	57.3 (12.1)	38.4	12	HbA_1c_^e^	Physician; dietitian, medical assistant; medical students; nurse; pharmacy students; physician assistant students (5)	Daily huddles, collaborative care plans, team visits, patient phone call follow-up, medication reconciliations, and student-led group diabetic visit guidelines; colocation; face-to-face communication
Provost et al,^59^ 2017 Canada	Retrospective pre-post	Health and social services center + collaboration with PCPs	1689	58.5	43.1	12	HbA_1c_^e^	Physician; kinesiologist; nurse; nutritionist; pharmacist; social worker (6)	Joint or group educational sessions; shared medical appointments or group visits; shared EMR; regularly scheduled meetings with interdisciplinary teams
							SBP^e^		
							DBP^e^		
Singh-Franco et al,^60^ 2013 United States	Retrospective pre-post	Mobile clinic	114	63 (11)	15	12	HbA_1c_^e^	Physician; nurse; nutritionist; pharmacist specialist in ambulatory care; translator (5)	Colocation; face-to-face communication^f^^,^^g^^,^^i^^,^^j^
							SBP		
							Statin use		
Watts et al,^61^ 2015 United States	Retrospective pre-post	Primary care clinic within Veterans Affairs	1170	62.6 (9.1)	96	6	HbA_1c_^e^	Physician; CDE (either an NP or a clinical pharmacy specialist); general internist; health psychologist; registered dietitian (4)	Shared medical appointments or group visits; colocation; face-to-face communication^f^^,^^j^
Zwar et al,^62^ 2007 Australia	Retrospective pre-post^n^	Health maintenance organization ambulatory care clinics	230	61.2 (11.4)	50.4	12	HbA_1c_^e^	Physician; diabetes educator; dietitian; optometrist; podiatrist (5)	Shared EMRs^j^
							SBP^e^		
							DBP^e^		

Abbreviations: ACIC, assessment of chronic illness care; ACO, accountable care organization; ASCVD, atherosclerotic cardiovascular disease; BH, behavioral health; C, comparison; CDE, board certified diabetes educator; CHW, community health worker; CPA, collaborative practice agreement; CV, cardiovascular; DBP, diastolic blood pressure; DCCP, diabetes care coordination program; DDMP, diabetes disease management program; EMPOWER–PAR, engaging and motivating patients online with enhanced resources-participatory action research; EMR, electronic medical record; FBG, fasting blood glucose; FQCHC, federally qualified community health center; HAPPY, health awareness and primary prevention in your neighborhood; HbA_1c_, hemoglobin A_1C_; I, intervention; ICP, interprofessional collaborative practice; IP, interprofessional; MEDIC-E, multidisciplinary education and diabetes intervention for cardiac risk reduction–extended; NP, nurse practitioner; PACT, patient-aligned care team; PCMH, patient-centered medical home; PCP, primary care professional; RCT, randomized clinical trial; SBP, systolic blood pressure; VA-MEDIC, Veterans Affairs Multi-disciplinary Education and Diabetes Intervention for Cardiac risk reduction.

^a^ Total number of enrolled patients; the number in analysis (Study N) are reported in forest plots (Figure 2, Figure 3, eMethod, and eFigure 1 in the Supplement).

^b^ First team member listed represents the primary care professional who served the gatekeeper functions of the “primary care provider.”

^c^ Data reported descriptively as each manuscript described/defined the interprofessional team/features/processes and based on the predetermined data extraction categories used in this meta-analysis.

^d^ Facility level.

^e^ An outcome that was included in the meta-analysis.

^f^ Patient education/counseling.

^g^ Health promotion/disease prevention.

^h^ Patient level.

^i^ Medication management.

^j^ Chronic disease management.

^k^ Adherence support.

^l^ Village level.

^m^ The study analyzed HbA_1c_ change from baseline to 18 and 36 months; the 18-month data was used in the meta-analysis.

^n^ Pre-post study comparing 2 independent groups before and after the intervention (before-and-after study).

### Study Outcomes

#### Hemoglobin A_1C_

**Figure 2.**
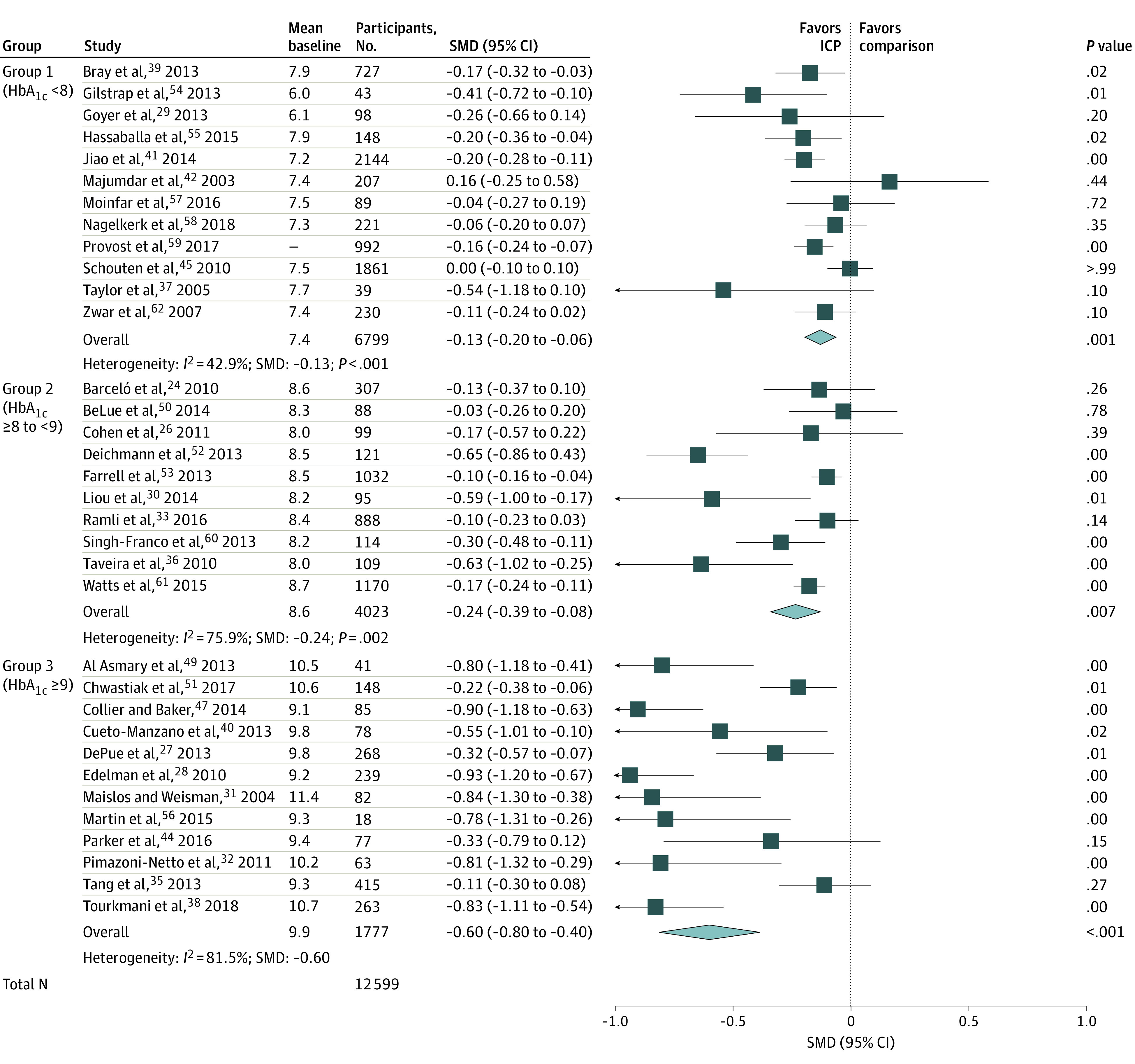
Association of Interprofessional Collaborative Practice (ICP) and Hemoglobin A_1c_ (HbA_1c_). Stratified by Baseline HbA_1c_ No overall standardized mean difference (SMD) was calculated because of the differences between the baseline HbA_1c_ groups. Squares represent mean values, with the size of the squares indicating weight and horizontal lines representing 95% CIs. Diamonds represent the pooled mean with the points of the diamonds representing 95% CIs.

In data pooled from 34 studies (N = 12 599) shown in Figure 2, ICP was associated with reduced HbA_1c_ for all groups regardless of baseline HbA_1c_ levels, although the SMD varied between the groups. For group 1 (mean baseline HbA_1c_, 7.4), the SMD was small at −0.13 (95% CI, −0.20 to −0.06; *P* < .001); for group 2 (mean baseline HbA_1c_, 8.6), the SMD was borderline moderate at −0.24 (95% CI, −0.39 to −0.08; *P* = .007); and for group 3 (mean baseline HbA_1c_, 9.9), the SMD was large at −0.60 (95% CI, −0.80 to −0.40; *P* < .001). The SMD was significantly greater for group 3 than for either group 1 (*P* < .001) or group 2 (*P* = .002), but the SMDs for group 2 and group 1 did not differ (*P* = .08). The SMD increased 80% from group 1 to group 2 and 250% from group 2 to group 3. Given the substantial differences among these groups, no overall SMD was calculated. Heterogeneity (*I*^2^) also varied in group 1 (*I*^2^ = 42.9%), group 2 (*I*^2^ = 79.9%), and group 3 (*I*^2^ = 81.5%), indicating significant between-study variations. In the leave-one-out analysis, removal of 1 study^52^ in group 2 reduced the group SMD by 27% from −0.24 to −0.17, which would have contributed to the heterogeneity of group 2. No other study changed group SMDs more than 18%. Heterogeneity was not associated with the number of professions involved in ICP; the correlation between the number of professions and decrease in HbA_1c_ was not significant. The correlation of study duration and HbA_1c_ effects was also nonsignificant.

The association of ICP with HbA_1c_ differed by study design (overall *P* = .03 for differences between the 3 types of studies) (eFigure 1 in the Supplement). The SMD was greatest for RCTs (SMD = −0.46; 95% CI, −0.65 to −0.27; *P* < .001), less for pre-post studies (SMD = −0.26; 95% CI, −0.40 to −0.12; *P* = .002), and least for prospective cohort studies (SMD = −0.14; 95% CI, −0.33 to −0.05; *P* = .11). Only the RCTs and prospective cohort studies differed significantly (*P* = .007), with no statistical difference between the RCTs and pre-post studies (*P* = .12) or pre-post studies and prospective cohort studies (*P* = .08). However, the research design was confounded by baseline HbA_1c_ levels. The mean baseline HbA_1c_ level for the prospective cohort studies was 7.5%; for pre-post studies, 8.4%; and for RCTs, 9.1%; which is similar to the baseline HbA_1c_ levels and the SMDs for HbA_1c_ reduction. In the funnel plot (eFigure 2 in the Supplement), missing studies in the right lower quadrant were noted, and Kendall τ rank correlation was significant (τ=−.37; *P* = .002), indicating likely publication bias. The fail-safe N = 2068 suggested that 2068 studies showing no effect are needed to reduce the SMD to 0.

#### Systolic Blood Pressure

**Figure 3.**
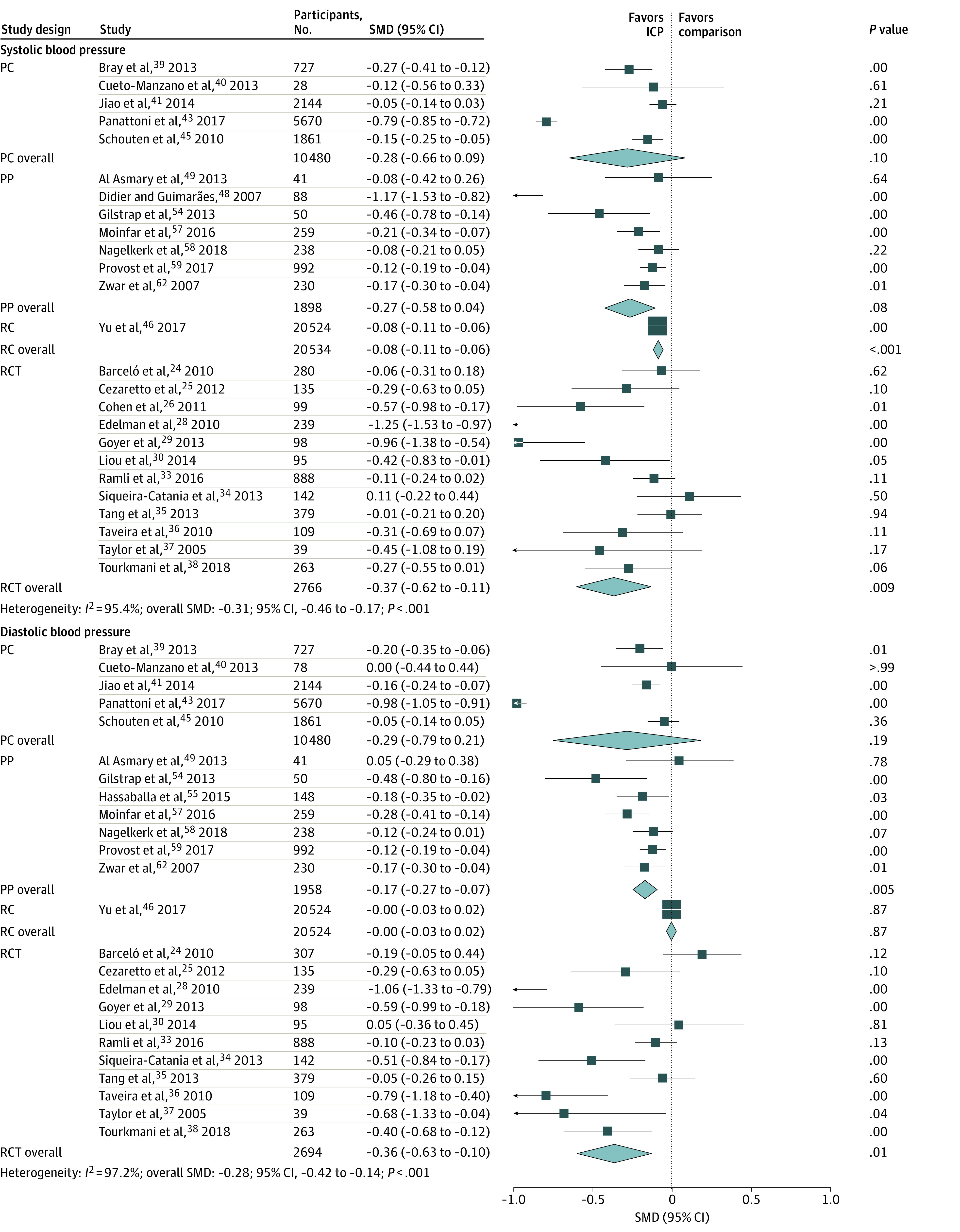
Association of Interprofessional Collaborative Practice (ICP) With Systolic Blood Pressure and Diastolic Blood Pressure, Stratified by Study Design Squares represent mean values, with the size of the squares indicating weight and horizontal lines representing 95% CIs. Diamonds represent the pooled mean with the points of the diamonds representing 95% CIs. PC indicates prospective cohort study; PP, pre-post study; RC, retrospective cohort study; RCT, randomized clinical trial; SMD, standard mean difference.

In data pooled from 25 studies (N = 35 618), shown in Figure 3, ICP was associated with a moderate effect on SBP; the overall SMD was −0.31 (95% CI, −0.46 to −0.17; *P* < .001). However, the SMD varied by study design. The SMD was significant for ICP in RCTs (SMD = −0.37; 95% CI, −0.62 to −0.11; *P* = .009) and the retrospective cohort study (SMD = −0.08; 95% CI, −0.11 to −0.06; *P* < .001) but not for prospective cohort studies (SMD = −0.28; 95% CI, −0.66 to −0.09; *P* = .10) or pre-post studies (SMD = −0.27; 95% CI, −0.58 to −0.04; *P* = .08). The SMD for the retrospective cohort study was significantly smaller than the SMDs for RCTs (*P* = .02) and pre-post studies (*P* = .02) but not statistically different from the SMD for prospective cohort studies (*P* = .29). Nonetheless, when excluding the retrospective cohort study, there was no difference in the SMD between RCTs, pre-post studies, and prospective cohort studies. Heterogeneity among the studies was high (*I*^2^ = 95.4% overall). Heterogeneity was also high among within-design groups: prospective cohort studies (*I*^2^ = 98.2%), RCTs (*I*^2^ = 86.4%), and pre-post studies (*I*^2^ = 84.1%). In the leave-one-out analysis, removal of 1 study^43^ decreased the overall SMD by 23%, contributing to heterogeneity. The SMD was not associated with baseline SBP levels (for SBP<130 vs SBP≥130; *P* = .76). The funnel plot (eFigure 3 in the Supplement) showed missing studies to the right of the mean. The Kendall τ rank correlation between SMD and SE was significant (τ=.22; *P* = .008), indicating likely publication bias. The fail-safe N was 1812 studies.

#### Diastolic Blood Pressure

In data pooled from 24 studies (N = 35 606), shown in Figure 3, ICP was associated with a moderate effect on DBP; the overall SMD was −0.28 (95% CI, −0.42 to −0.14; *P* < .001). However, the SMD varied by study design. The SMD was significant for ICP in the RCTs (SMD = −0.36, 95% CI, −0.63 to −0.10; *P* = .01) and pre-post studies (SMD = −0.17; 95% CI, −0.27 to −0.07; *P* = .005) but not in the prospective cohort studies (SMD = −0.29, 95% CI, −0.79 to 0.21; *P* = .19) or retrospective cohort study (SMD = 0.00, 95% CI, −0.03 to 0.03; *P* = .87). The SMD for the retrospective cohort study was significantly smaller than the SMDs for the RCTs (*P* = .006) and pre-post studies (*P* < .001) but not statistically different from the SMD for prospective cohort studies (*P* = .39). Nevertheless, there was no difference between the SMDs for the RCTs, pre-post studies, and prospective cohort studies (*P* = .31). Heterogeneity was high among the prospective studies (*I*^2^ = 98.9%; *P* < .001) and RCTs (*I*^2^ = 86.1%; *P* < .001) but not among the pre-post studies (*I*^2^ = 39.7%; *P* = .13). In the leave-one-out analysis, the removal of 1 study^43^ reduced the SMD by 24%, contributing to the heterogeneity. The SMD was not associated with baseline DBP levels (for DBP<80 vs DBP≥80; *P* = .45). No publication bias was noted; the funnel plot showed no missing studies (eFigure 4 in the Supplement), and the Kendall τ rank correlation was nonsignificant (τ=.22; *P* = .14). The fail-safe N was 1539 studies.

### Bias Assessment

The bias assessment for studies included in the meta-analyses are presented in Table 2. Overall, RCTs scored a low risk for most factors, but there was a mixed unclear and high-risk majority for “knowledge of allocated interventions” (n = 9) and “contamination” (n = 8). The non-RCT studies showed most high-risk scores for “allocation sequence generation” (n = 21) and “concealment of allocation” (n = 18) and mixed unclear and high-risk scores for “dropouts, attrition” (n = 19) and “knowledge of allocated interventions” (n = 14).

**Table 2.** Risk of Bias Assessment for Studies Included in the Meta-analysis

Source	Allocation sequence generation	Concealment of allocation	Equivalence at baseline		Dropouts, attrition	Knowledge of allocated interventions	Contamination	Selective reporting	Intervention fidelity
			Outcome	Group					
**Randomized clinical trials**									
Barceló et al,^24^ 2010	Unclear	Low	Low	Low	Low	Unclear	Low	Low	Unclear
Cezaretto et al,^25^ 2012	Low	Unclear	Low	Low	Low	Unclear	Unclear	Low	Low
Cohen et al,^26^ 2011	Low	Unclear	Low	Low	Low	Unclear	Unclear	Low	Low
DePue et al,^27^ 2013	Low	Low	Low	Unclear	Low	Unclear	Low	Low	Low
Edelman et al,^28^ 2010	Unclear	Unclear	Unclear	High	Low	Low	Unclear	Low	Low
Goyer et al,^29^ 2013	Low	Low	Low	Low	Low	Unclear	Unclear	Low	Low
Liou et al,^30^ 2014	Unclear	Unclear	Low	Low	Unclear	Low	Low	Low	Low
Maislos and Weisman,^31^ 2004	Low	Low	High	High	High	Unclear	Low	Low	Low
Pimazoni-Netto et al,^32^ 2011	Unclear	Unclear	Low	High	Unclear	Unclear	Unclear	Low	Low
Ramli et al,^33^ 2016	Low	Low	Low	Low	Low	Low	Low	Low	Low
Siqueira-Catania et al,^34^ 2013	Unclear	Unclear	High	High	Unclear	Low	Unclear	Low	Low
Tang et al,^35^ 2013	Low	Low	Low	Low	High	Low	Unclear	Low	Low
Taveira et al,^36^ 2010	Low	Unclear	High	High	High	Unclear	High	Low	Low
Taylor et al,^37^ 2005	Low	Low	Low	Unclear	Low	Unclear	Low	Low	Low
Tourkmani et al,^38^ 2018	High	Low	Unclear	Low	Unclear	Low	Low	Unclear	Low
**Prospective cohort studies**									
Bray et al,^39^ 2013	Low	Low	Low	Low	High	High	Low	Low	Low
Cueto-Manzano et al,^40^ 2013	High	Low	Unclear	Low	Unclear	Low	Low	Unclear	Low
Jiao et al,^41^ 2014	High	High	Low	Low	Low	Unclear	Unclear	Low	Unclear
Majumdar et al,^42^ 2003	Low	Low	Low	Low	High	High	Low	Low	Low
Panattoni et al,^43^ 2017	High	Unclear	Low	High	Unclear	High	Low	Low	Low
Parker et al,^44^ 2016	High	High	Unclear	High	High	Low	Unclear	Low	Unclear
Schouten et al,^45^ 2010	High	Low	Low	High	Low	High	Low	Low	Unclear
**Retrospective cohort studies**									
Yu et al,^46^ 2017	High	High	Low	Low	Unclear	Low	Low	Low	Low
**Prospective pre-post studies**									
Collier and Baker,^47^ 2014	High	High	Low	Low	High	Low	Unclear	Low	Low
Didier and Guimarães,^48^ 2007	High	High	Low	Low	High	High	High	Low	Unclear
**Retrospective pre-post studies**									
Al Asmary et al,^49^ 2013	High	High	Low	Low	Low	High	Low	Low	Low
BeLue et al,^50^ 2014	High	High	Low	High	Unclear	Unclear	Unclear	Low	Unclear
Chwastiak et al,^51^ 2017	High	High	High	High	Unclear	Low	Unclear	Low	Low
Deichmann et al,^52^ 2013	Low	Unclear	High	High	High	Low	Unclear	Low	Low
Farrell et al,^53^ 2013	High	High	Unclear	High	Low	High	Low	Low	Low
Gilstrap et al,^54^ 2013	High	High	Low	Low	High	High	Low	High	Low
Hassaballa et al,^55^ 2015	High	High	Low	Low	High	Unclear	Low	Low	Low
Martin et al,^56^ 2015	High	High	Low	Low	High	Low	Low	Low	Low
Moinfar et al,^57^ 2016	High	High	Low	Low	Low	High	Low	Low	Unclear
Nagelkerk et al,^58^ 2018	High	High	Low	Low	Unclear	Low	Low	Low	Low
Provost et al,^59^ 2017	High	High	High	High	High	Low	Low	Low	Low
Singh-Franco et al,^60^ 2013	High	High	Low	Low	Unclear	High	Low	Low	Unclear
Watts et al,^61^ 2015	High	High	Low	Low	Unclear	Low	Low	Low	Low
Zwar et al,^62^ 2007	High	High	Unclear	Unclear	High	High	Unclear	Low	High

Abbreviations: High, high risk of bias; Low, low risk of bias; Unclear, unclear risk of bias.

## Discussion

A notable finding from the current meta-analysis (n = 39) is that ICP was associated with reduced HbA_1c_ levels regardless of the baseline HbA_1c_ level and decreased SBP and DBP in adult primary care patients with diabetes and/or hypertension. The ICP effect estimate was substantial for patients with a baseline HbA_1c_ greater than or equal to 9 (250% larger than the effect estimate for baseline HbA_1c_≥8 to <9), but no correlation was found between baseline BP levels and ICP. Although ICP teams (≥3 different professions) delivered varied interventions within diverse primary care settings, the association was significantly positive across all SMDs, with the largest effect size for the highest baseline HbA_1c_ group and a moderate effect size for both SBP and DBP. For HbA_1c_, 2068 negative studies are needed to negate the favorable effects by ICP. For SBP and DBP, important clinical measures of hypertension and cardiovascular status for diabetes, 1812 and 1539 negative studies, respectively, are needed to refute the effects of ICP.

To our knowledge, this is the most up-to-date and inclusive systematic review and meta-analysis on ICP in primary care for patients with diabetes and/or hypertension (50 studies in systematic review and 39 in meta-analysis). While previous research has assessed the association between team care and diabetes and hypertension outcomes, the latest search, to our knowledge, ended in 2015 in an RCT-only meta-analysis.^6^ Conducted in controlled environments involving specified patient populations and using precise interventions, RCTs have a superior study design with a lower risk of bias. Yet, the findings from RCTs may lack real-life scenarios and patient behaviors in response to clinical interventions that more closely reflect everyday experience. Moreover, previous research included teams of at least 2 professionals in various settings, whereas we included ICPs of at least 3 health professions in primary care. Among the 35 studies in the 2019 meta-analysis,^6^ only 2 studies overlapped with the 39 studies included in our meta-analysis,^27,37^ indicating differences in research scope.

To strengthen the confidence to detect the directly aligned effects of ICP, we strictly adhered to the prespecified inclusion criteria and required the use of explicitly stated data from each study. Therefore, in study selection, we excluded studies that did not clearly report involvement of at least 3 professions in primary care. For example, a study of pharmacists working with physicians and other health care professionals on patients with diabetes that provided no specification for “other providers” was excluded.^74^ Further, we excluded studies with outcome measures reported in a format that was not suitable for SMD calculation from the meta-analysis. For bias assessment, we used tools specific for rating RCTs and non-RCTs and found RCTs appraised as having a lower risk of bias compared with non-RCTs.

Heterogeneity was substantial for all of the outcomes (HbA_1c_, SBP, and DBP). For HbA_1c_, baseline HbA_1c_ likely contributed to the heterogeneity, but significant heterogeneity remained within the HbA_1c_ groups. For SBP and DBP, we found no association between baseline BP levels and BP reduction; however, the heterogeneity was high. Study design may have been a factor in the heterogeneity, but it was difficult to assess for HbA_1c_ given the confounding by baseline HbA_1c_ levels. The BP stratification by study design revealed significant differences in overall SMD for SBP and DBP, with RCTs and prospective studies showing larger effect sizes compared with the other designs. Such differences may stem from studies with more control having the intervention group receive all aspects of the intervention, whereas less controlled studies may have missing intervention aspects or contaminated comparison groups. The number of professions included in the ICP teams did not seem to contribute to the heterogeneity. The study duration also varied (3-24 months), yet the association of study duration and HbA_1c_ was not significant. Hence, heterogeneity may be associated with factors that were not assessed in this meta-analysis, such as intervention dose-effect.

Sources of variation were also likely due to differences in sample size and population, setting, and possible publication bias. Sample size may have similar effects as the study design; for example, smaller studies may be easier to control than very large studies. Simultaneously, studies with a small sample size may have been underpowered to detect the intervention effect, and biased selection may have taken place. There was a varying degree of diabetes control among the participants indicated by baseline HbA_1c_ levels, which may mean that the source populations were varied. Although the mean age ranged from 51 to 70 years, only 2 studies reported a mean age greater than 65 years. While all ICP teams delivered primary care (18 in the US and 21 elsewhere), study settings varied from ambulatory care clinics to community health centers, public health centers, Veterans Affairs health systems. and other settings, with differing resources and infrastructures for ICP provision. Publication bias, which can also be a factor in variation among included studies, was found to be likely for HbA_1c_ and SBP.

Similar to previous findings,^21,75,76^ we uncovered inconsistencies among the number and types of professionals involved in ICP, how the team functioned, and types of interventions delivered. The number of professions ranged from 3 to 10, which suggests differing interventions delivered by diverse expertise. The focus of our study, however, was to assess ICP and not the addition of specific health care professionals. The secondary analysis showed no association between the number of professions in ICP and HbA_1c_ reduction. The teamwork and communication strategies varied, although colocation was most often reported (n = 30), followed by having shared electronic medical records (n = 10) and weekly or biweekly team meetings (n = 7). Regarding the interventions, 13 teams provided joint/group educational sessions and 11 had shared/group visits. With such diversity, identifying an ideal team feature and function for effectiveness and efficiency, perhaps tailored to patient risk, may be an appropriate future research area.

### Limitations

This study has limitations. No determination of differences in the source population was evaluated, such as educational level that may be a factor in medication adherence, lifestyle modifications that can affect outcomes, or insurance information that may reveal socioeconomic status. Neither the degree of integration among team members in primary care nor the intervention intensity was clearly specified in most studies. Study funding sources were also not considered. Despite these limitations, we assessed an ample number of studies that used the equivalent outcome measures. Worldwide, health care is transforming rapidly, with team-based care suggested for diverse patients. Concurrently, aging populations with chronic conditions may overwhelm primary care systems. ICP appears to be a plausible option for areas with limited access to care and in patients with poorer diabetes control. Using our findings, primary care practices may wish to consider providing ICP involving at least 3 professions to improve diabetes and hypertension outcomes.

## Conclusions

The results of this systematic review and meta-analysis suggest that there is a positive association of ICP in primary care with HbA_1c_, SBP, and DBP levels in adult patients with diabetes or hypertension. Adults with diabetes and/or hypertension should receive team-based care to improve outcomes.

## Notes

Supplement. **eMethods 1.** Search Strategy for Ovid MEDLINE ALL (1946 to March 02, 2018) **eMethods 2.** Meta-analysis Supplemental Materials **eTable.** Characteristics of the Studies Not Included in the Meta-analysis **eFigure 1.** Association of ICP With Hemoglobin A_1C_ Stratified by Study Design **eFigure 2.** Funnel Plot for Hemoglobin A_1C_ **eFigure 3.** Funnel Plot for Systolic Blood Pressure **eFigure 4.** Funnel Plot for Diastolic Blood Pressure
