# Error in Introduction

**DOI:** 10.1001/jamanetworkopen.2021.9114

**Published:** 2021-04-09

In the Original Investigation titled “Assessment of Interprofessional Collaborative Practices and Outcomes in Adults With Diabetes and Hypertension in Primary Care: A Systematic Review and Meta-analysis,”^1^ published February 12, 2021, there was an error in the Introduction. Two sentences in the third paragraph of the Introduction mischaracterized a meta-analysis as not including a systematic review. The sentences should have appeared as “A 2019 systematic review and meta-analysis of 35 studies reported that, compared with usual care, team-based care was associated with improved HbA_1c_, systolic blood pressure (SBP), and diastolic blood pressure (DBP) levels.[6] This study included randomized clinical trials (RCTs) only up to 2015 and was not focused on assessing ICP by at least 3 professions in primary care settings.” This article has been corrected.^1^
