# Supplementary material for: Assessment of Interprofessional Collaborative Practices and Outcomes in Adults With Diabetes and Hypertension in Primary Care: A Systematic Review and Meta-analysis
Source: JAMA Netw Open. 2021 Feb 12;4(2):e2036725. doi: 10.1001/jamanetworkopen.2020.36725 (PMC7881360; doi:10.1001/jamanetworkopen.2020.36725)
Supplement: Supplement. — eMethods 1. Search Strategy for Ovid MEDLINE ALL (1946 to March 02, 2018) eMethods 2. Meta-analysis Supplemental Materials eTable. Characteristics of the Studies Not Included in the Meta-analysis eFigure 1. Association of ICP With Hemoglobin A1C Stratified by Study Design eFigure 2. Funnel Plot for Hemoglobin A1C eFigure 3. Funnel Plot for Systolic Blood Pressure eFigure 4. Funnel Plot for Diastolic Blood Pressure [file jamanetwopen-e2036725-s001.pdf]

Supplemental Online Content

Lee JK, McCutcheon LRM, Fazel MT, Cooley JH, Slack MK. Assessment of interprofessional collaborative practices and putcomes in adults with diabetes and hypertension in primary care: a systematic review and meta-analysis. *JAMA Netw Open*. 2021;4(2):e2036725. doi:10.1001/jamanetworkopen.2020.36725

eMethods 1. Search Strategy for Ovid MEDLINE ALL (1946 to March 02, 2018)
eMethods 2. Meta-analysis Supplemental Materials
eTable. Characteristics of the Studies Not Included in the Meta-analysis
eFigure 1. Association of ICP With A1C Stratified by Study Design
eFigure 2. Funnel Plot for Hemoglobin A1C
eFigure 3. Funnel Plot for Systolic Blood Pressure
eFigure 4. Funnel Plot for Diastolic Blood Pressure

This supplemental material has been provided by the authors to give readers additional information about their work.

© 2021 Lee JK et al. *JAMA Network Open*.

eMethods 1. Search strategy for Ovid MEDLINE(R) ALL (1946 to March 02, 2018).

Search conducted March 5, 2018.

1	exp Primary Health Care/
2	General Practice/
3	Family Practice/
4	General Practitioners/
5	Physicians, Primary Care/
6	Physicians, Family/
7	Primary Care Nursing/
8	Community Health Services/
9	Ambulatory Care Facilities/
10	Ambulatory Care/
11	Internal Medicine/
12	internal medicine.tw.
13	((primary or ambulatory or community) adj3 care).tw.
14	((primary or ambulatory or community) adj3 healthcare).tw.
15	((primary or ambulatory or community) adj3 health service$).tw.
16	(primary adj3 provider$).tw.
17	((general or ambulatory or primary or family) adj3 pract$).tw.
18	generalist$.tw.
19	gp.tw.
20	gps.tw.
21	family health$.tw.
22	family medicine.tw.
23	family physician$.tw.
24	family doctor$.tw.
25	aboriginal health centre$.tw.
26	community health center$.tw.
27	community health centre$.tw.
28	centre local de services.tw.
29	clscs.tw.
30	communautaire$.tw.
31	community service center$.tw.
32	community service centre$.tw.
33	(nurse adj3 led clinic$).tw.
34	outpost nursing station$.tw.
35	or/1-34

© 2021 Lee JK et al. *JAMA Network Open*.

36	Patient Care Team/
37	Cooperative Behavior/
38	interprofessional relations/
39	interdisciplinary communication/
40	physician-nurse relations/
41	Group Processes/
42	interprofessional$.af.
43	inter-professional$.af.
44	interdisciplinary.tw.
45	inter-disciplinary.tw.
46	multidisciplinary.tw.
47	multi-disciplinary.tw.
48	transdisciplinary.tw.
49	trans-disciplinary.tw.
50	cross-disciplinary.tw.
51	multiprofessional$.tw.
52	multi-professional$.tw.
53	transprofessional$.tw.
54	trans-professional$.tw.
55	team$.tw.
56	collaborat$.tw.
57	shared care.tw.
58	(shared adj3 appointment$).tw.
59	or/36-58
60	triple aim.tw.
61	(35 and 59) or 60
62	exp Hypertension/
63	hypertens$.tw.
64	Blood Pressure/
65	blood pressure.tw.
66	bloodpressure.tw.
67	Prehypertension/
68	prehypertension.tw.
69	systolic.tw.
70	diastolic.tw.
71	exp DIABETES MELLITUS/
72	diabet$.tw.
73	glucose intolerance.tw.

© 2021 Lee JK et al. *JAMA Network Open*.

74	insulin resistance.tw.
75	IDDM.tw.
76	NIDDM.tw.
77	T2DM.tw.
78	T1DM.tw.
79	or/62-78
80	61 and 79
81	limit 80 to yr="2013 -Current"
82	limit 81 to english language

© 2021 Lee JK et al. *JAMA Network Open*.

eMedthods 2. Meta-analysis supplemental materials

The general procedure used was to enter the data into CMA spreadsheet based on the data reported. The tabs at the bottom of the page provide information on the types of data formats that can be used. For any study reporting CI's without SD's, the SD was calculated from the CI interval using the following procedure: Calculate total width of CI, divide by 3.92, and multiply by the square root of N.

The data was used from only one arm in 2 studies, Chwastiak, 2017, and Deichmann, 2013 and were classified as pre-post studies.

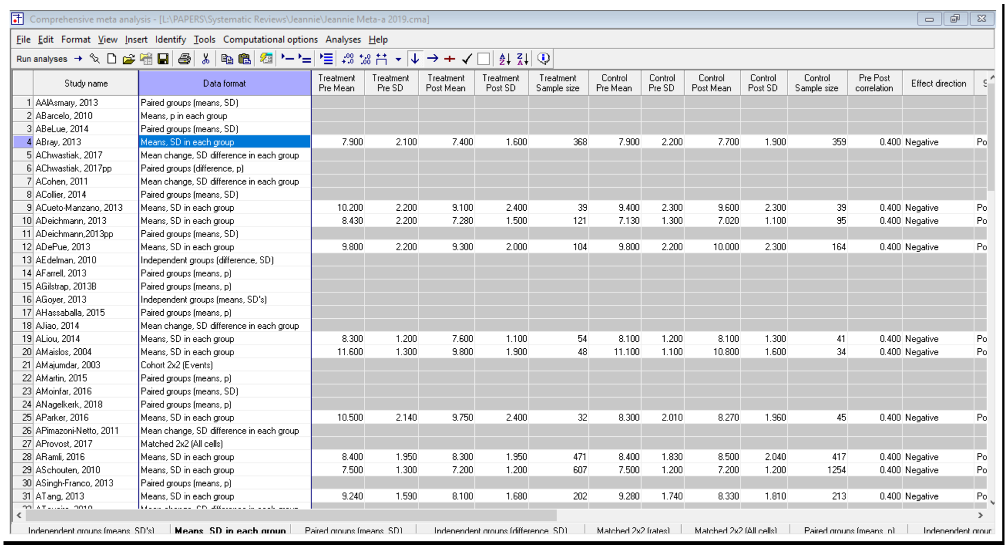

	Study name	Data format	Treatment Pre Mean	Treatment Pre SD	Treatment Post Mean	Treatment Post SD	Treatment Sample size	Control Pre Mean	Control Pre SD	Control Post Mean	Control Post SD	Control Sample size	Pre Post correlation	Effect direction	S
1	AAlAsmary, 2013	Paired groups (means, SD)													
2	ABarcelo, 2010	Means, p in each group													
3	ABeLue, 2014	Paired groups (means, SD)													
4	ABray, 2013	Means, SD in each group	7.900	2.100	7.400	1.600	368	7.900	2.200	7.700	1.900	359	0.400	Negative	Po
5	AChwastiak, 2017	Mean change, SD difference in each group													
6	AChwastiak, 2017pp	Paired groups (difference, p)													
7	ACohen, 2011	Mean change, SD difference in each group													
8	ACollier, 2014	Paired groups (means, SD)													
9	ACueto-Manzano, 2013	Means, SD in each group	10.200	2.200	9.100	2.400	39	9.400	2.300	9.600	2.300	39	0.400	Negative	Po
10	ADeichmann, 2013	Means, SD in each group	8.430	2.200	7.280	1.500	121	7.130	1.300	7.020	1.100	95	0.400	Negative	Po
11	ADeichmann,2013pp	Paired groups (means, SD)													
12	ADePue, 2013	Means, SD in each group	9.800	2.200	9.300	2.000	104	9.800	2.200	10.000	2.300	164	0.400	Negative	Po
13	AEdelman, 2010	Independent groups (difference, SD)													
14	AFarrell, 2013	Paired groups (means, p)													
15	AGilstrap, 2013B	Paired groups (means, p)													
16	AGoyer, 2013	Independent groups (means, SD's)													
17	AHassaballa, 2015	Paired groups (means, p)													
18	AJiao, 2014	Mean change, SD difference in each group													
19	ALiou, 2014	Means, SD in each group	8.300	1.200	7.600	1.100	54	8.100	1.200	8.100	1.300	41	0.400	Negative	Po
20	AMaislos, 2004	Means, SD in each group	11.600	1.300	9.800	1.900	48	11.100	1.100	10.800	1.600	34	0.400	Negative	Po
21	AMajumdar, 2003	Cohort 2x2 (Events)													
22	AMartin, 2015	Paired groups (means, p)													
23	AMoinfar, 2016	Paired groups (means, SD)													
24	ANagelkerk, 2018	Paired groups (means, p)													
25	AParker, 2016	Means, SD in each group	10.500	2.140	9.750	2.400	32	8.300	2.010	8.270	1.960	45	0.400	Negative	Po
26	APimazoni-Netto, 2011	Mean change, SD difference in each group													
27	AProvost, 2017	Matched 2x2 (All cells)													
28	ARamli, 2016	Means, SD in each group	8.400	1.950	8.300	1.950	471	8.400	1.830	8.500	2.040	417	0.400	Negative	Po
29	ASchouten, 2010	Means, SD in each group	7.500	1.300	7.200	1.200	607	7.500	1.200	7.200	1.200	1254	0.400	Negative	Po
30	ASingh-Franco, 2013	Paired groups (means, p)													
31	ATang, 2013	Means, SD in each group	9.240	1.590	8.100	1.680	202	9.280	1.740	8.330	1.810	213	0.400	Negative	Po

The next step was to select the model to be used, random or fixed, and examine the data output to be used for the forest plot.

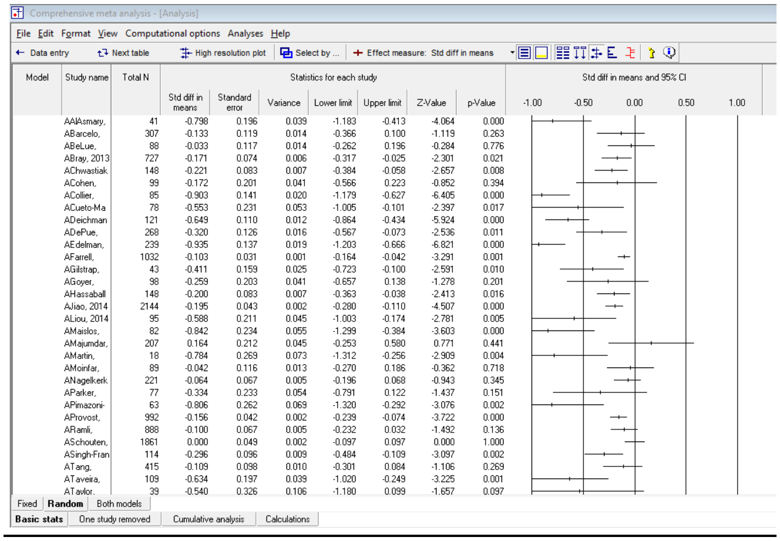

Model	Study name	Total N	Std diff in means	Standard error	Variance	Lower limit	Upper limit	Z-Value	p-Value
	AAlAsmary,	41	-0.798	0.196	0.039	-1.183	-0.413	-4.064	0.000
	ABarcelo,	307	-0.133	0.119	0.014	-0.366	0.100	-1.119	0.263
	ABeLue,	88	-0.033	0.117	0.014	-0.262	0.196	-0.284	0.776
	ABray, 2013	727	-0.171	0.074	0.006	-0.317	-0.025	-2.301	0.021
	AChwastiak	148	-0.221	0.083	0.007	-0.384	-0.058	-2.657	0.008
	ACohen,	99	-0.172	0.201	0.041	-0.566	0.223	-0.852	0.394
	ACollier,	85	-0.903	0.141	0.020	-1.179	-0.627	-6.405	0.000
	ACueto-Ma	78	-0.553	0.231	0.053	-1.005	-0.101	-2.397	0.017
	ADeichman	121	-0.649	0.110	0.012	-0.864	-0.434	-5.924	0.000
	ADePue,	268	-0.320	0.126	0.016	-0.567	-0.073	-2.536	0.011
	AEdelman,	239	-0.935	0.137	0.019	-1.203	-0.666	-6.821	0.000
	AFarrell,	1032	-0.103	0.031	0.001	-0.164	-0.042	-3.291	0.001
	AGilstrap,	43	-0.411	0.159	0.025	-0.723	-0.100	-2.591	0.010
	AGoyer,	98	-0.259	0.203	0.041	-0.657	0.138	-1.278	0.201
	AHassaball	148	-0.200	0.083	0.007	-0.363	-0.038	-2.413	0.016
	AJiao, 2014	2144	-0.195	0.043	0.002	-0.280	-0.110	-4.507	0.000
	ALiou, 2014	95	-0.588	0.211	0.045	-1.003	-0.174	-2.781	0.005
	AMaislos,	82	-0.842	0.234	0.055	-1.299	-0.384	-3.603	0.000
	AMajumdar,	207	0.164	0.212	0.045	-0.253	0.580	0.771	0.441
	AMartin,	18	-0.784	0.269	0.073	-1.312	-0.256	-2.909	0.004
	AMoinfar,	89	-0.042	0.116	0.013	-0.270	0.186	-0.362	0.718
	ANagelkerk	221	-0.064	0.067	0.005	-0.196	0.068	-0.943	0.345
	AParker,	77	-0.334	0.233	0.054	-0.791	0.122	-1.437	0.151
	APimazoni-	63	-0.806	0.262	0.069	-1.320	-0.292	-3.076	0.002
	AProvost,	992	-0.156	0.042	0.002	-0.239	-0.074	-3.722	0.000
	ARamli,	888	-0.100	0.067	0.005	-0.232	0.032	-1.492	0.136
	ASchouten,	1861	0.000	0.049	0.002	-0.097	0.097	0.000	1.000
	ASingh-Fran	114	-0.296	0.096	0.009	-0.484	-0.109	-3.097	0.002
	ATang,	415	-0.109	0.098	0.010	-0.301	0.084	-1.106	0.269
	ATaveira,	109	-0.634	0.197	0.039	-1.020	-0.249	-3.225	0.001
	ATaylor,	39	-0.540	0.326	0.106	-1.180	0.099	-1.657	0.097

The moderator variable was identified (baseline A1C group) and applied to the analysis.

No mean baseline mean HA1c was provided in Provost, 2017. The study was included in the <8 group because 63% of the sample had an HA1c less than 7. A weighted mean for baseline A1C was calculated for each A1C group.

© 2021 Lee JK et al. *JAMA Network Open*.

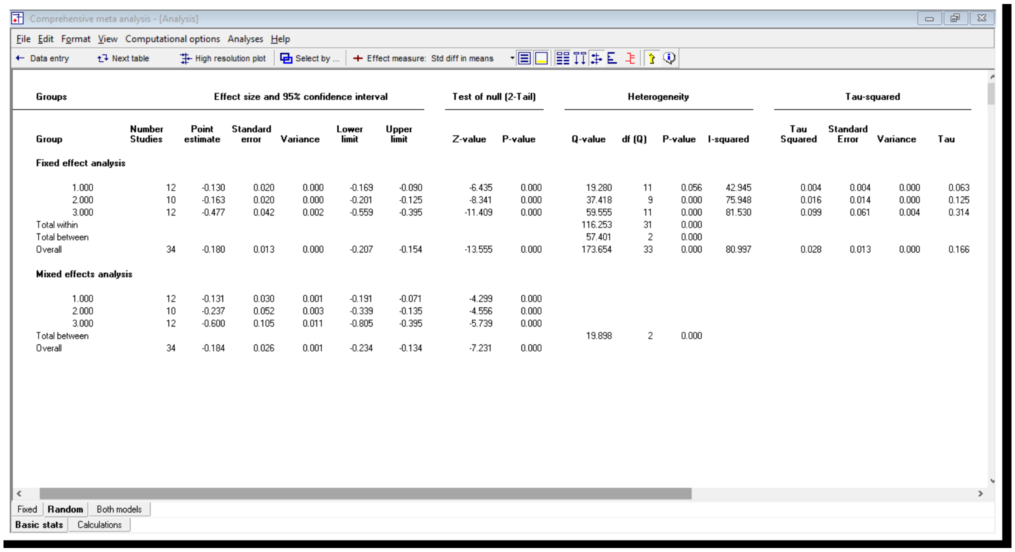

Comprehensive meta analysis - [Analysis]

Groups		Effect size and 95% confidence interval					Test of null (2-Tail)		Heterogeneity				Tau-squared			
Group	Number Studies	Point estimate	Standard error	Variance	Lower limit	Upper limit	Z-value	P-value	Q-value	df (Q)	P-value	I-squared	Tau Squared	Standard Error	Variance	Tau
**Fixed effect analysis**																
1.000	12	-0.130	0.020	0.000	-0.169	-0.090	-6.435	0.000	19.280	11	0.056	42.945	0.004	0.004	0.000	0.063
2.000	10	-0.163	0.020	0.000	-0.201	-0.125	-8.341	0.000	37.418	9	0.000	75.948	0.016	0.014	0.000	0.125
3.000	12	-0.477	0.042	0.002	-0.559	-0.395	-11.409	0.000	59.555	11	0.000	81.530	0.099	0.061	0.004	0.314
Total within									116.253	31	0.000					
Total between									57.401	2	0.000					
Overall	34	-0.180	0.013	0.000	-0.207	-0.154	-13.555	0.000	173.654	33	0.000	80.997	0.028	0.013	0.000	0.166
**Mixed effects analysis**																
1.000	12	-0.131	0.030	0.001	-0.191	-0.071	-4.299	0.000								
2.000	10	-0.237	0.052	0.003	-0.339	-0.135	-4.556	0.000								
3.000	12	-0.600	0.105	0.011	-0.805	-0.395	-5.739	0.000								
Total between									19.898	2	0.000					
Overall	34	-0.184	0.026	0.001	-0.234	-0.134	-7.231	0.000								

The HKSJ adjustment was used to correct the confidence intervals for each group. No overall SMD was calculated for A1C given that patients with different baseline A1C levels represent different populations.

The forest plot was generated.

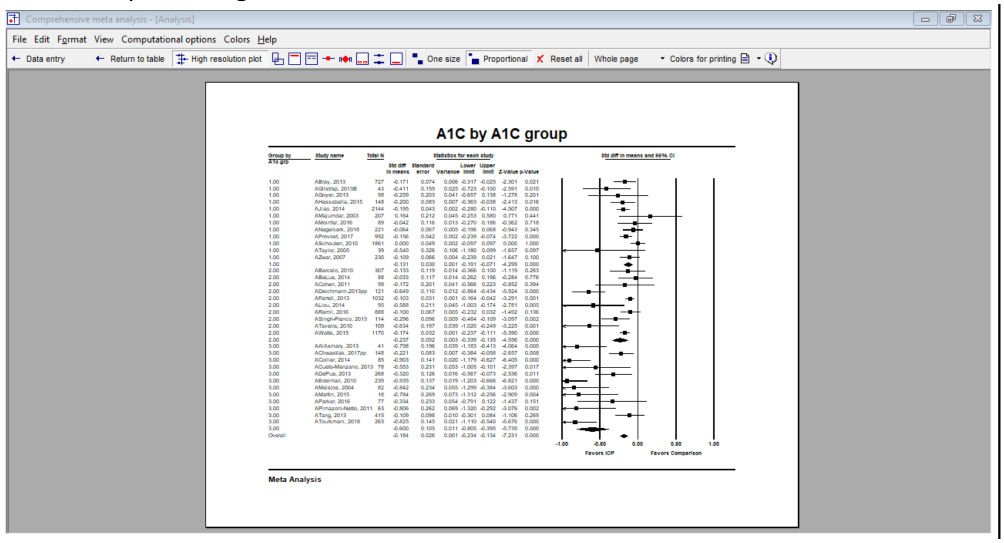

After generation of the forest plot, other analyses were generated using the non-stratified data, for example, the funnel plot, fail-safe N, and Kendall's tau. CI's and p-values were corrected using the HKSJ procedure and changed in the Word version of the forest plot.

Reference:

IntHout J, Ioannidis JPA, Borm GF. The Hartung-Knapp-Sidik-Jonkman method for random effects meta-aanlysis is straightforward and considerably outperforms the standard DerSimonian-Laird method. BMC Medical Research Methodology. 2014; 14:25.

© 2021 Lee JK et al. *JAMA Network Open*.

eTable. Characteristics of the studies not included in the meta-analysis

Source	Study design	Setting	Total No.[a]	Age mean (SD), y	% Male	Duration, months	Outcome measures	Team members (No. of professions in team)[b]	Main ICP team features or process (name of intervention program/model if specified OR other notable specifics)[c]
**Randomized clinical trials (RCTs)**									
Hutchison et al,[1] 2014 United States	RCT[d]	Volunteer driven community health center	124	Not reported	Not reported	I: 24 + follow-up at 36	HbA_{1c}<br>SBP<br>DBP	Physician/doctor of nursing practice; clinical pharmacist; NP; physician; students (pre-medical, pharmacy, nursing)	Students had team building activities; co-location
Katon et al,[2] 2004 United States	RCT[d]	Ambulatory care clinics	329	I: 58.6 (11.8)<br>C: 58.1 (12.0)	I: 34.<br>C: 35.2	12	HbA_{1c}	Physician; nurse; psychologist; psychiatrist	CPA; co-location; shared EMR; face-to-face (Individualized-stepped-care depression treatment program)[e,f,g]
Lin et al,[3] 2014 United States	RCT[d]	Group health primary care clinics	214	I: 57.4 (10.5)<br>C: 56.3 (12.1)	I: 52<br>C: 44	12 + follow-up at 12	HbA_{1c}<br>SBP	Physician; medical consultant; patients; psychiatric consultant; TEAMcare nurse care managers	Weekly case reviews; shared EMRs; co-location; face-to-face communication[e,f,g,h]
**Prospective cohort studies**									
Tobe et al,[4] 2014 Canada	Prospective cohort	Ambulatory care clinic/ center/ office	2855	64.5 (12.1)	44	9	SBP<br>DBP	Physician; community pharmacists; nurse; NP	IP group educational sessions; shared EMRs; regular team meetings, teleconferences, email; face-to-face communication; practice-based tools[e,f,g,h]
**Retrospective cohort studies**									
De La Rosa et al,[5] 2020 United States	Retrospective cohort	Family Practice Center	119	I: 58<br>C: 59	Not reported	12	HbA_{1c}<br>BP	Physician; medical assistants; medical students; nursing students	Interactive teamwork led by attending physician with nursing students serving as case managers
Reiss-Brennan et al,[6] 2016 United States	Retrospective cohort	Health Systems Primary Care Clinics	113 452	56.1	39.81	36	HbA_{1c}<br>SBP<br>DBP	Primary care physician; clinic managers; medical support staff; mental health specialists; nurse care managers	Shared EMR; co-location (also established protocols, knowledge of team roles, use of decision support tools, standard assessment.)

© 2021 Lee JK et al. *JAMA Network Open*.

**Prospective pre-post studies**									
Fortuna,[7] 2015 United States	Prospective pre-post	Urban internal medicine practice	13 404	Not reported	44.8	42	BP	Physician; pharmacist; nurse	Co-management by pharmacist (patient and physician education, self-management for medication adherence) and RN (intensive self-management education, BP monitoring, reports to physicians)[f,i]
**Retrospective pre-post studies**									
Otero-Sabogal et al,[8] 2010 United States	Retrospective pre-post	Community Health Center	114	35	30.70	Up to 24	HbA_{1c}<br>SBP<br>DBP	Physician; CHW; social worker; CDEs (profession unspecified)	Group patient educational sessions; Transforming primary care practice to PCMH; shared-medical appointments or group visits; co-location; face-to-face communication (Healthfirst Model)[e,f,g,h,i]
Rossom et al,[9] 2017 United States	Retrospective pre-post	Group-model; 18 care systems and 172 clinics in rural, urban, suburban settings	3609	60 (12)	62	Mean (range): 11 (1-26)	HbA_{1c}<br>SBP<br>DBP	Physician; care manager; psychiatrist	Weekly meeting for case reviewsj; co-location; telecommunication/telemedicine; electronic care management tracking system
Edwards et al,[10] 2019 United States	Retrospective pre-post	Veteran Affairs primary care clinics	44 527	I: 59.3 (15.2)<br>C: 61.8 (15.3)	I: 83.6<br>C: 91.6	48	HbA_{1c}	Physician; NP; pharmacist; psychologist trainees	IP education; shared EMRs; co-location (CoEPCE, an initiative designed to promote IP education)
Hull et al,[11] 2014 United Kingdom	Retrospective pre-post[j]	Networks of Ambulatory care practices	41 210	Not reported	Not reported	36	HbA_{1c}	General Practitioner; care coordinator; community-based diabetes specialist nurses; consultant diabetologist	Joint IP or group patient educational sessions; shared EMRs; co-location[e,g,h]

Abbreviations: CDE, board certified diabetes educator; CoEPCE, centers of excellence in primary care education; CPA, collaborative practice agreement; DBP, diastolic blood pressure; EMR, electronic medical record; HbA_{1c}, hemoglobin A_{1c}; ICP, interprofessional collaborative practice; IP, interprofessional; NP, nurse practitioner; PCMH, patient-centered medical home; RCT, randomized clinical trial; RN, registered nurse, SBP, systolic blood pressure; SD, standard deviation; TEAMcare, treatment, enhancement, activation, and motivation care.

[a] Total number of enrolled patients.

[b] First team member listed represents the primary care professional who served the gatekeeper functions of the "primary care provider".

[c] Data reported descriptively as each manuscript described/defined the interprofessional team/features/processes and based on the predetermined data extraction categories used in this systematic review.

[d] Patient-level.

[e] Patient education/counseling.

[f] Medication management.

[g] Chronic disease management.

[h] Health promotion/disease prevention.

[I] Adherence support.

[j] Pre-post study comparing 2 independent groups before and after the intervention (before-and-after study).

© 2021 Lee JK et al. *JAMA Network Open*.

Hemoglobin A1C

Study Design	Study	Baseline A1C	Study N	Statistics for each study: Std diff in means	Upper limit	Lower limit	p-value
PC	Bray et al,[12] 2013	7.9	727	-0.17	-0.32	-0.03	0.02
	Cueto-Manzano et al,[13] 2013	9.8	78	-0.55	-1.01	-0.10	0.02
	Jiao et al,[14] 2014	7.2	2144	-0.20	-0.28	-0.11	0.00
	Majumdar et al,[15] 2003	7.4	207	0.16	-0.25	0.58	0.44
	Parker et al,[16] 2016	9.4	77	-0.33	-0.79	0.12	0.15
	Schouten et al,[17] 2010	7.5	1861	0.00	-0.10	0.10	1.00
PC Overall		7.5	5094	- 0.14	-0.33	-0.05	0.114
PP	Al Asmary et al,[18] 2013	10.5	41	-0.80	-1.18	-0.41	0.00
	BeLue et al,[19] 2014	8.3	88	-0.03	-0.26	0.20	0.78
	Chwastiak et al,[20] 2017	10.6	148	-0.22	-0.38	-0.06	0.01
	Collier et al,[21] 2014	9.1	85	-0.90	-1.18	-0.63	0.00
	Deichmann et al,[22] 2013	8.5	121	-0.65	-0.86	-0.43	0.00
	Farrell et al,[23] 2013	8.5	1032	-0.10	-0.16	-0.04	0.00
	Gilstrap et al,[24] 2013	6.0	43	-0.41	-0.72	-0.10	0.01
	Hassaballa et al,[25] 2015	7.9	148	-0.20	-0.36	-0.04	0.02
	Martin et al,[26] 2015	9.3	18	-0.78	-1.31	-0.26	0.00
	Moinfar et al,[27] 2016	7.5	89	-0.04	-0.27	0.19	0.72
	Nagelkerk et al,[28] 2018	7.3	221	-0.06	-0.20	0.07	0.35
	Provost et al,[29] 2017	---	992	-0.16	-0.24	-0.07	0.00
	Singh-Franco et al,[30] 2013	8.2	114	-0.30	-0.48	-0.11	0.00
	Watts et al,[31] 2015	8.7	1170	-0.17	-0.24	-0.11	0.00
	Zwar et al,[32] 2007	7.4	230	-0.11	-0.24	0.02	0.10
PP Overall		8.4	4540	-0.26	-0.40	-0.12	0.002
RCT	Barcelo et al,[33] 2010	8.6	307	-0.13	-0.37	0.10	0.26
	Cohen et al,[34] 2011	8.0	99	-0.17	-0.57	0.22	0.39
	DePue et al,[35] 2013	9.8	268	-0.32	-0.57	-0.07	0.01
	Edelman et al,[36] 2010	9.2	239	-0.93	-1.20	-0.67	0.00
	Goyer el al,[37] 2013	6.	98	-0.26	-0.66	0.14	0.20
	Liou et al,[38] 2014	8.2	95	-0.59	-1.00	-0.17	0.01
	Maislos et al,[39] 2004	11.4	82	-0.84	-1.30	-0.38	0.00
	Pimazoni-Netto et al,[40] 2011	10.2	63	-0.81	-1.32	-0.29	0.00
	Ramli et al,[41] 2016	8.4	888	-0.10	-0.23	0.03	0.14
	Tang et al,[42] 2013	9.3	415	-0.11	-0.30	0.08	0.27
	Taveira et al,[43] 2010	8.0	109	-0.63	-1.02	-0.25	0.00
	Taylor et al,[44] 2005	7.7	39	-0.54	-1.18	0.10	0.10
	Tourkmani et al,[45] 2018	10.7	263	-0.83	-1.11	-0.54	0.00
RCT Overall		9.1	2965	-0.46	-0.65	-0.27	<0.001
Total N			12599				

Std Diff in Means and 95% CI

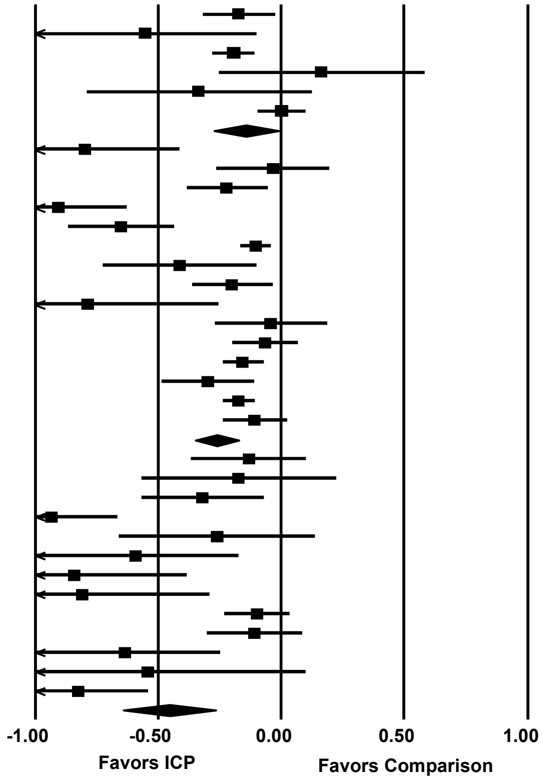

eFigure 1. Association of ICP with A1C stratified by study design

Std diff = standard difference; CI = confidence interval; ICP = Interprofessional Collaborative Practice; PC = prospective cohort studies; PP = pre-post studies; RC = retrospective cohort study; RCT = randomized control trial; Total N for SBP=35,668; Total N for DBP=35,656; the overall SMD for SBP=-0.31, 95% CI, -0.46 to -0.17, p<0.001; the overall SMD for DBP=-0.28, 95% CI, -0.42 to -0.14, p<0.001. For SBP, the SMD for the RC study was less than the SMD for PP studies (p=0.021) and RCTs (p=0.015) but not different from PC studies (p=0.286). For DBP, the SMD for RC study was statistically less than for PP studies (<0.001) and RCTs (p=0.006), but not different from PC studies (p=0.387). For both SBP and DBP there was no difference between the PC, PP, or RCT groups (p>0.270). Heterogeneity I^2=95.4% for SBP and 97.2% for DBP.

© 2021 Lee JK et al. *JAMA Network Open*.

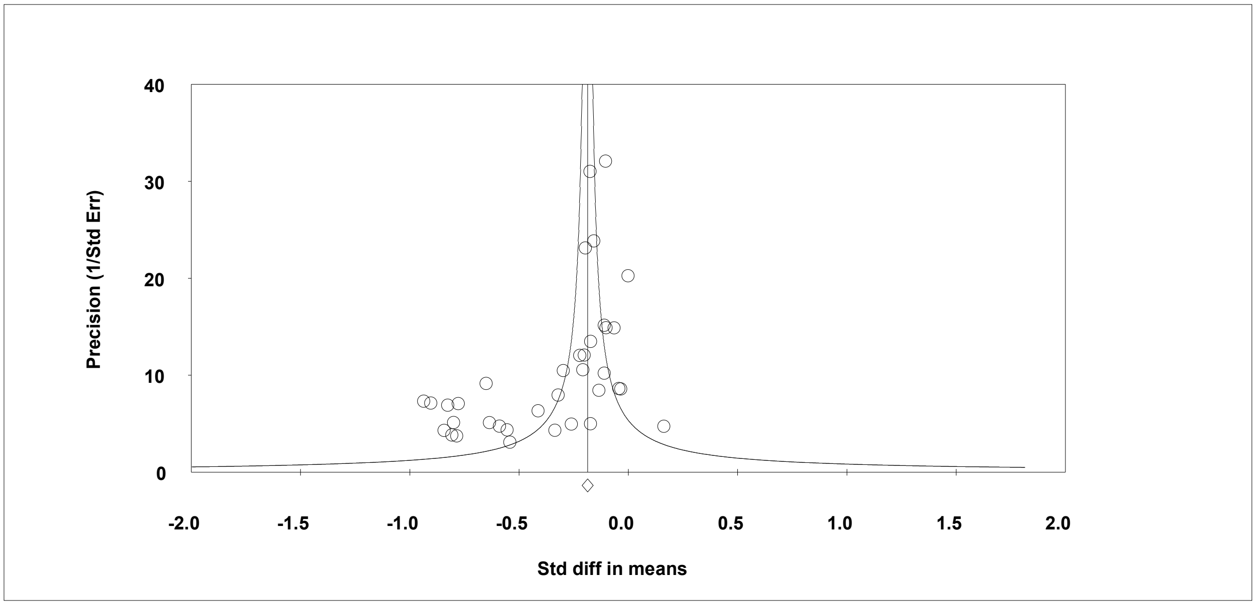

eFigure 2. Funnel plot for hemoglobin A1C
Std diff = standard difference; Std Err = standard error; The p-value for Kendall's tau was 0.002.

© 2021 Lee JK et al. *JAMA Network Open*.

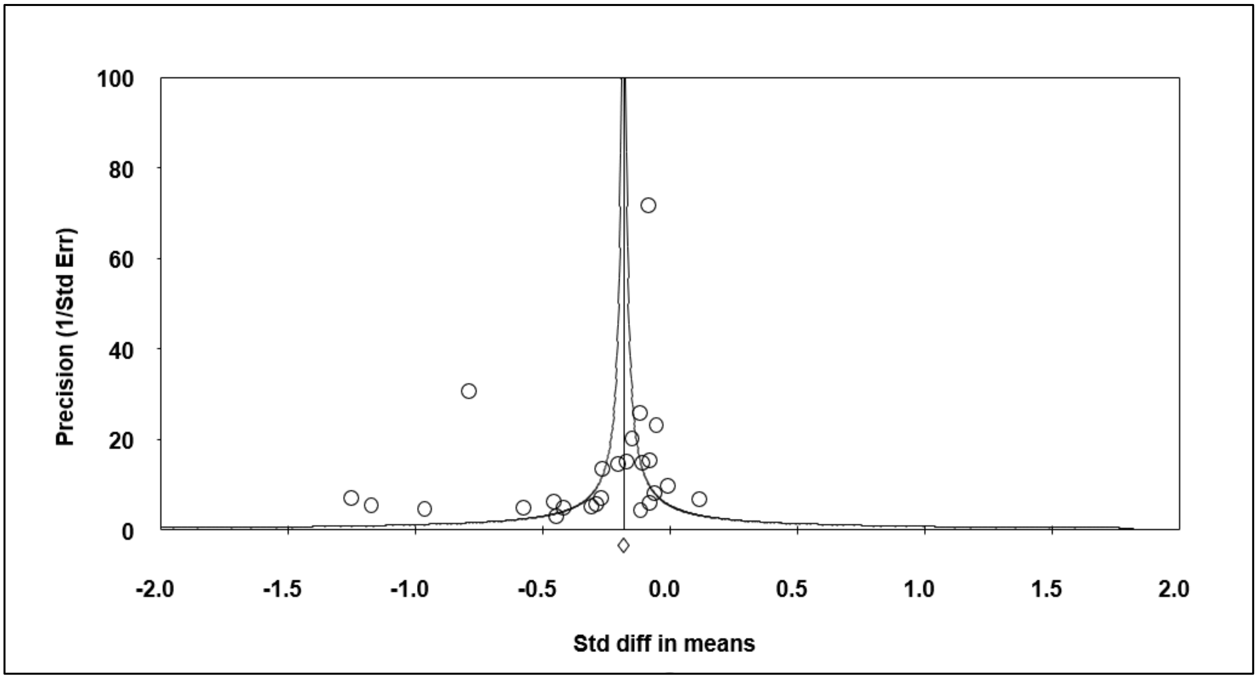

eFigure 3. Funnel plot for systolic blood pressure

Std diff = standard difference, Std Err = standard error. The p-value for Kendall's tau was 0.008, indicating that publication bias was likely present in this group of studies.

© 2021 Lee JK et al. *JAMA Network Open*.

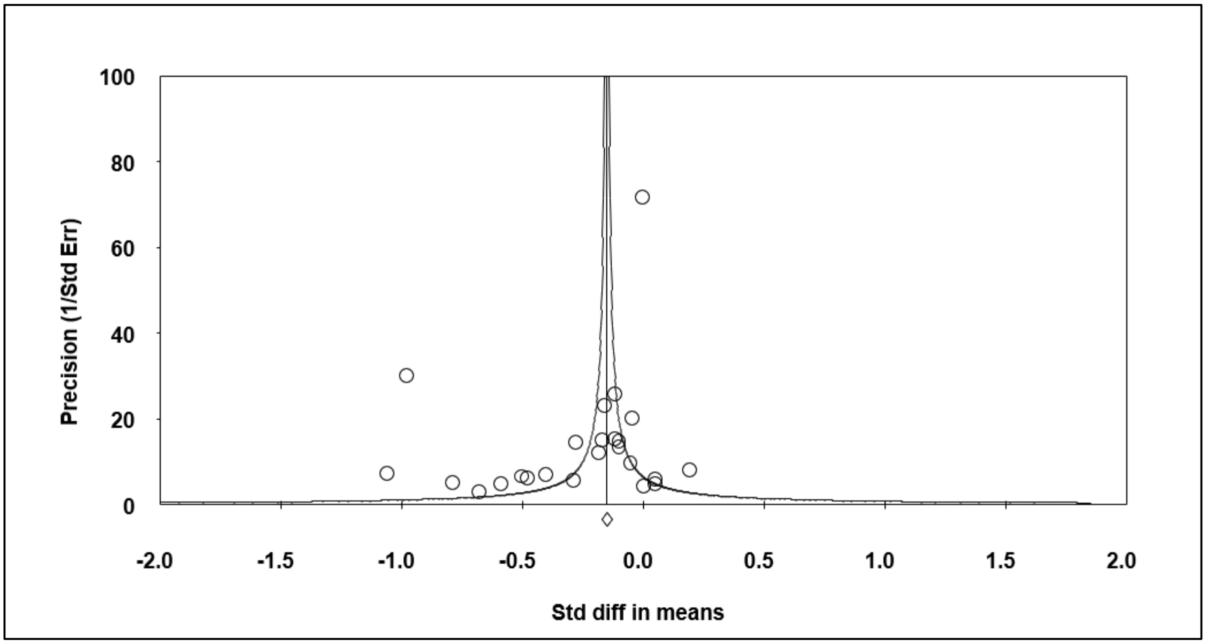

eFigure 4. Funnel plot for diastolic blood pressure

Std diff = standard difference, Std err = standard error. The p-value for Kendall's tau was 0.137, indicating that publication bias was not likely in this group of studies.

© 2021 Lee JK et al. *JAMA Network Open*.

eReferences

1 Hutchison RJ. Treating diabetes in underserved populations using an interprofessional care team. J Interprof Care. 2014;28(6):568-569.

2. Katon WJ, Von Korff M, Lin EH, et al. The Pathways Study: a randomized trial of collaborative care in patients with diabetes and depression. Arch Gen Psychiatry. 2004;61(10):1042-1049.

3. Lin EH, Von Korff M, Peterson D, Ludman EJ, Ciechanowski P, Katon W. Population targeting and durability of multimorbidity collaborative care management. Am J Manag Care. 2014;20(11):887-895.

4. Tobe SW, Moy Lum-Kwong M, Von Sychowski S, Kandukur K, Kiss A, Flintoft V. Hypertension management initiative prospective cohort study: comparison between immediate and delayed intervention groups. J Hum Hypertens. 2014;28(1):44-50.

5. De La Rosa M, Pitts S, Chen PH. An interprofessional collaboration of care to improve clinical outcomes for patients with diabetes. J Interprof Care. 2020;34(2):269-271.

6. Reiss-Brennan B, Brunisholz KD, Dredge C, et al. Association of integrated team-based care with health care quality, utilization, and cost. JAMA. 2016;316(8):826-834.

7. Fortuna RJ, Nagel AK, Rose E, et al. Effectiveness of a multidisciplinary intervention to improve hypertension control in an urban underserved practice. J Am Soc Hypertens. 2015;9(12):966-974.

8. Otero-Sabogal R, Arretz D, Siebold S, et al. Physician-community health worker partnering to support diabetes self-management in primary care. Qual Prim Care. 2010;18(6):363-372.

9. Rossom RC, Solberg LI, Magnan S, et al. Impact of a national collaborative care initiative for patients with depression and diabetes or cardiovascular disease. General Hospital Psychiatry. 2017;44:77-85.

10. Edwards ST, Hooker ER, Brienza R, et al. Association of a multisite interprofessional education initiative with quality of primary care. JAMA Netw Open. 2019;2(11):e1915943.

11. Hull S, Chowdhury TA, Mathur R, Robson J. Improving outcomes for patients with type 2 diabetes using general practice networks: a quality improvement project in east London. BMJ Quality & Safety. 2014;23(2):171-176.

12. Bray P, Cummings DM, Morrissey S, et al. Improved outcomes in diabetes care for rural African Americans. Ann Fam Med. 2013;11(2):145-150.

13. Cueto-Manzano AM, Martinez-Ramirez HR, Cortes-Sanabria L. Comparison of primary health-care models in the management of chronic kidney disease. Kidney International Supplements. 2013;3(2):210-214.

14. Jiao FF, Fung CS, Wong CK, et al. Effects of the multidisciplinary Risk Assessment and Management Program for Patients With Diabetes Mellitus (RAMP-DM) on biomedical outcomes, observed cardiovascular events and cardiovascular risks in primary care: a longitudinal comparative study. Cardiovasc Diabetol. 2014;13:127.

15. Majumdar SR, Guirguis LM, Toth EL, Lewanczuk RZ, Lee TK, Johnson JA. Controlled trial of a multifaceted intervention for improving quality of care for rural patients with type 2 diabetes. Diabetes Care. 2003;26(11):3061-3066.

16. Parker RA, Hook LD, Jones ME. Glycemic control: can nurse practitioners on interprofessional collaborative practice teams enhance clinical outcomes? J Am Assoc Nurse Pract. 2016;28(12):652-658.

17. Schouten LM, Niessen LW, van de Pas JW, Grol RP, Hulscher ME. Cost-effectiveness of a quality improvement collaborative focusing on patients with diabetes. Med Care. 2010;48(10):884-891.

18. Al Asmary SM, Al-Harbi T, Tourkmani AM, et al. Impact of integrated care program on glycemic control and cardiovascular risk in adult patients with type 2 diabetes. J Clin Outcomes Manage. 2013;20(8):356-363.

19. Belue R, Figaro MK, Peterson J, Wilds C, William P. The diabetes healthy outcomes program: results of free health care for uninsured at a federally qualified community health center. J Prim Care Community Health. 2014;5(1):4-8.

20. Chwastiak LA, Jackson SL, Russo J, et al. A collaborative care team to integrate behavioral health care and treatment of poorly-controlled type 2 diabetes in an urban safety net primary care clinic. Gen Hosp Psychiatry. 2017;44:10-15.

21. Collier IA, Baker DM. Implementation of a pharmacist-supervised outpatient diabetes treatment clinic. Am J Health-Syst Pharm. 2014;71(1):27-36.

22. Deichmann RE, Hebert AM, Harmeyer ED, Cazabon P, Chavis E. Effects of a diabetes boot cAMP on hemoglobin a1c levels. Ochsner J. 2013;13(2):194-199.

23. Farrell P, Barnaby S, Galarza T, et al. Population management of diabetes in a high-need urban community in the Bronx: the experience of Montefiore Medical Center. Diabetes Educator. 2013;39(4):515-522.

24. Gilstrap LG, Malhotra R, Peltier-Saxe D, et al. Community-based primary prevention programs decrease the rate of metabolic syndrome among socioeconomically disadvantaged women. J Womens Health (Larchmt). 2013;22(4):322-9.

© 2021 Lee JK et al. *JAMA Network Open*.

25. Hassaballa I, Ebekozien O, Ogungbadero A, et al. Evaluation of a diabetes care coordination program for African-American women living in public housing. J Clin Outcomes Manag. 2015;22(8):365-372.
26. Martin SL, Williams E, Huerth B, Robinson JD; Penobscot Nation Health Center's Diabetes Team. A pharmacy student-facilitated interprofessional diabetes clinic with the Penobscot Nation. Prev Chronic Dis. 2015;12:E190.
27. Moinfar Z, Sedaghat M, Abolhassani F, et al. A collaborative care program for management of common mental disorders among diabetic patients in a primary healthcare setting. J Pub Health (Germany). 2016;24(4):273-279.
28. Nagelkerk J, Thompson ME, Bouthillier M, et al. Improving outcomes in adults with diabetes through an interprofessional collaborative practice program. J Interprofessional Care. 2018;32(1):4-13.
29. Provost S, Pineault R, Grimard D, et al. Implementation of an integrated primary care cardiometabolic risk prevention and management network in Montreal: does greater coordination of care with primary care physicians have an impact on health outcomes? Health Promotion and Chronic Disease Prevention in Canada. 2017;37(4):105-113.
30. Singh-Franco D, Perez A, Wolowich WR. Improvement in surrogate endpoints by a multidisciplinary team in a mobile clinic serving a low-income, immigrant minority population in South Florida. J Health Care Poor Underserved. 2013 Feb;24(1):67-77.
31. Watts SA, Strauss GJ, Pascuzzi K, et al. Shared medical appointments for patients with diabetes: glycemic reduction in high-risk patients. J Am Assoc Nurse Pract. 2015;27(8):450-456.
32. Zwar NA, Hermiz O, Comino EJ, Shortus T, Burns J, Harris M. Do multidisciplinary care plans result in better care for patients with type 2 diabetes? Aust Fam Physician. 2007;36(1-2):85-89.
33. Barceló A, Cafiero E, de Boer M, et al. Using collaborative learning to improve diabetes care and outcomes: the VIDA project. Prim Care Diabetes. 2010;4(3):145-153.
34. Cohen LB, Taveira TH, Khatana SA, Dooley AG, Pirraglia PA, Wu WC. Pharmacist-led shared medical appointments for multiple cardiovascular risk reduction in patients with type 2 diabetes. Diabetes Educ. 2011;37(6):801-812.
35. DePue JD, Dunsiger S, Seiden AD, et al. Nurse-community health worker team improves diabetes care in American Samoa: results of a randomized controlled trial. Diabetes Care. 2013;36(7):1947-1953.
36. Edelman D, Fredrickson SK, Melnyk SD, et al. Medical clinics versus usual care for patients with both diabetes and hypertension: a randomized trial. Ann Intern Med. 2010;152(11):689-696.
37. Goyer L, Dufour R, Janelle C, et al. Randomized controlled trial on the long-term efficacy of a multifaceted, interdisciplinary lifestyle intervention in reducing cardiovascular risk and improving lifestyle in patients at risk of cardiovascular disease. J Behav Med. 2013;36(2):212-224.
38. Liou JK, Soon MS, Chen CH, et al. Shared care combined with telecare improves glycemic control of diabetic patients in a rural underserved community. Telemed J E Health. 2014;20(2):175-178.
39. Maislos M, Weisman D. Multidisciplinary approach to patients with poorly controlled type 2 diabetes mellitus: a prospective, randomized study. Acta Diabetol. 2004;41(2):44-48.
40. Pimazoni-Netto A, Rodbard D, Zanella MT. Rapid improvement of glycemic control in type 2 diabetes using weekly intensive multifactorial interventions: structured glucose monitoring, patient education, and adjustment of therapy-a randomized controlled trial. Diabetes Technol Ther. 2011;13(10):997-1004.
41. Ramli AS, Selvarajah S, Daud MH, et al. Effectiveness of the EMPOWER-PAR intervention in improving clinical outcomes of type 2 diabetes mellitus in primary care: a pragmatic cluster randomised controlled trial. BMC Fam Pract. 2016;17(1):157.
42. Tang PC, Overhage JM, Chan AS, et al. Online disease management of diabetes: engaging and motivating patients online with enhanced resources-diabetes (EMPOWER-D), a randomized controlled trial. J Am Med Inform Assoc. 2013;20(3):526-534.
43. Taveira TH, Friedmann PD, Cohen LB, et al. Pharmacist-led group medical appointment model in type 2 diabetes. Diabetes Educ. 2010;36(1):109-117.
44. Taylor KI, Oberle KM, Crutcher RA, Norton PG. Promoting health in type 2 diabetes: nurse-physician collaboration in primary care. Biol Res Nurs. 2005;6(3):207-215.
45. Tourkmani AM, Abdelhay O, Alkhashan HI, et al. Impact of an integrated care program on glycemic control and cardiovascular risk factors in patients with type 2 diabetes in Saudi Arabia: an interventional parallel-group controlled study. BMC Family Practice. 2018;19(1):1.

© 2021 Lee JK et al. *JAMA Network Open*.
